# Fueling the Firefighter and Tactical Athlete with Creatine: A Narrative Review of a Key Nutrient for Public Safety

**DOI:** 10.3390/nu16193285

**Published:** 2024-09-28

**Authors:** Drew E. Gonzalez, Scott C. Forbes, Annette Zapp, Andrew Jagim, Joel Luedke, Broderick L. Dickerson, Alexandria Root, Adriana Gil, Sarah E. Johnson, Macilynn Coles, Allison Brager, Ryan J. Sowinski, Darren G. Candow, Richard B. Kreider

**Affiliations:** 1 Exercise and Sport Nutrition Laboratory, Department of Kinesiology and Sport Management, Texas A&M University, College Station, TX 77843, USA; dickersobl5@tamu.edu (B.L.D.); sjohnson2216@tamu.edu (S.E.J.); rjs370@tamu.edu (R.J.S.); rbkreider@tamu.edu (R.B.K.); 2 Tactical Athlete Research Unit, Texas A&M University, College Station, TX 77843, USA; macilynn.coles@tamu.edu; 3 Department of Physical Education Studies, Faculty of Education, Brandon University, Brandon, MB R7A 6A9, Canada; forbess@brandonu.ca; 4 Fire Rescue Wellness, Guthrie, OK 73044, USA; info@firerescuewellness.org; 5 Sports Medicine, Mayo Clinic Health System, La Crosse, WI 54601, USA; jagim.andrew@mayo.edu; 6 Olmsted Medical Center-Sports Medicine, La Crosse, WI 54601, USA; jluedke@olmmed.org; 7 Rooted in Dietetics LLC, Taylor, MI 48180, USA; abrenay@emich.edu; 8 College of Medicine, University of Houston, Houston, TX 77021, USA; agil4@cougarnet.uh.edu; 9 U.S. Army John F. Kennedy Special Warfare Center and School, Fort Liberty, NC 48397, USA; allison.j.brager.mil@socom.mil; 10 Faculty of Kinesiology and Health Studies, University of Regina, Regina, SK S4S 0A2, Canada; darren.candow@uregina.ca

**Keywords:** firefighting, supplementation, tactical athletes, tactical professions ergogenic, performance, brain health

## Abstract

**Background/Objectives:** Firefighters, tactical police officers, and warriors often engage in periodic, intermittent, high-intensity physical work in austere environmental conditions and have a heightened risk of premature mortality. In addition, tough decision-making challenges, routine sleep deprivation, and trauma exacerbate this risk. Therefore, identifying strategies to bolster these personnel’s health and occupational performance is critical. Creatine monohydrate (CrM) supplementation may offer several benefits to firefighters and tactical athletes (e.g., police, security, and soldiers) due to its efficacy regarding physical performance, muscle, cardiovascular health, mental health, and cognitive performance. **Methods:** We conducted a narrative review of the literature with a focus on the benefits and application of creatine monohydrate among firefighters. **Results:** Recent evidence demonstrates that CrM can improve anaerobic exercise capacity and muscular fitness performance outcomes and aid in thermoregulation, decision-making, sleep, recovery from traumatic brain injuries (TBIs), and mental health. Emerging evidence also suggests that CrM may confer an antioxidant/anti-inflammatory effect, which may be particularly important for firefighters and those performing tactical occupations exposed to oxidative and physiological stress, which can elicit systemic inflammation and increase the risk of chronic diseases. **Conclusions:** This narrative review highlights the potential applications of CrM for related tactical occupations, with a particular focus on firefighters, and calls for further research into these populations.

## 1. Introduction

Firefighting’s physical, mental, and environmental demands induce considerable physiological stress (e.g., 70–73% VO_2max_ and ≈90% heart rate maximum) [1,2]. Similarly, law enforcement and military personnel face intense physical and mental occupational demands [2]. Therefore, these personnel require well-developed cardiorespiratory and musculoskeletal systems to meet their occupational demands [1,2,3,4,5]. Beyond the physical characteristics, the hazardous environmental conditions and thermoregulatory strain induce oxidative stress (OS) and inflammation and exacerbate chronic disease risk (e.g., cardiovascular disease [CVD]) [1,3,6,7,8,9,10,11,12,13]. Unfortunately, poor lifestyle factors (e.g., poor dietary habits and sleep loss) are prevalent and associated with job-related stress [2]. Thus, pragmatic strategies are needed to support firefighters and related tactical occupations in terms of health and occupational performance.

One dietary strategy to enhance physical and mental health is creatine monohydrate (CrM) supplementation, an efficacious and popular ergogenic aid [14,15]. In particular, CrM supplementation increases intramuscular creatine and phosphocreatine (PCr) concentrations and improves exercise performance and training adaptations [14]. Creatine monohydrate supplementation appears to play a role in post-exercise recovery, injury prevention, thermoregulation, and neuroprotection [14,16], with minimal risk of adverse events [14]. Emerging evidence suggests that CrM supplementation can enhance cognitive function, particularly when the brain is stressed (e.g., sleep loss, mental fatigue, and following a traumatic brain injury [TBI]) [17,18,19]. While the use of creatine among firefighters is unknown, it is plausible that firefighters have similar usage rates to the military [20,21]. These potential benefits can improve firefighters’ performance, readiness, health, and other tactical and occupational athletic groups (e.g., military and law enforcement). Thus, while this narrative review focuses on firefighters, the discussed benefits of CrM supplementation can be leveraged across populations with similar challenge patterns (e.g., intense physical, mental, and environmental job demands).

## 2. Materials and Methods

Given the increased interest in firefighter nutrition and the purported benefits of creatine supplementation [2], this narrative review aims to (1) highlight how CrM supplementation can improve firefighter health and performance, (2) provide practical recommendations, and (3) outline current gaps and future directions. Relevant literature included in this narrative review was searched via PubMed, Google Scholar, and fire department websites. Given that occupational demand is similar to other related tactical professions, we have also included literature regarding the application of creatine usage and insight into the context in which creatine may aid these other tactical personnel. Published articles between 1952 and June 2024 were considered for inclusion in this narrative review. Keywords utilized in the searches for creatine and its benefits, as well as firefighter-specific articles, included the following: “creatine”, creatine monohydrate”, “body composition”, fat mass”, lean mass”, “bone”, “exercise”, “exercise performance”, “thermoregulation”, “vascular health”, “concussion”, “firefighter”, “fire service”, “mental stress”, “resistance training”, “tactical performance”, “cardiovascular disease”, “heart attack”, “sudden cardiac event”, “obesity”, “inflammation”, “oxidative stress”, “physiological stress”, “disease”, “cardiorespiratory fitness”, “VO_2max_”, “nutrition”, “dietary intervention”, “dietary supplementation”, “shift work”, “sleep latency”, “disrupted sleep”, “self-contained breathing apparatus”, “personal protective equipment”, “heat stress”, “occupational exposures”, “suicide”, “psychological”, “post-traumatic stress disorder”, and “mental health”, “core temperature”, “thermoregulation”, “heat”, “thermal strain”, “sweat rate”, “sweat loss”, and “body water.” Non-English manuscripts were excluded from this review.

## 3. Overview of Creatine and Creatine Metabolism

Creatine (N-(aminoiminomethyl)-N-methyl glycine) is a naturally occurring pleiotropic compound synthesized endogenously from glycine, arginine, and methionine or obtained through the consumption of animal protein [14,22]. Post-ingestion and absorption, creatine enters the bloodstream and is transported to various storage sites, such as the muscle, brain, and testes. On average, a 70 kg male will have around 90–160 mmol/kg of total creatine stored in skeletal muscle [23]. Increasing muscle creatine content significantly increases PCr, which is vital to replenishing and maintaining adenosine triphosphate (ATP) levels during high-intensity exercise.

In healthy individuals, about half of the daily need for creatine is obtained via creatine synthesis, while the remaining amount is obtained in the diet, primarily from meat and fish. Creatine monohydrate supplementation (containing 87.9% creatine by molecular weight) is creatine bound ionically to water that readily dissociates during digestion [22]. Studies show that CrM supplementation increases free creatine, PCr, and total creatine content typically by 20–40% [22]. The purest and most well-studied, cost-effective, and internationally approved source of supplemental creatine is CrM from Germany [22,24]. Creatine monohydrate is nearly 100 percent bioavailable [22], and confers its benefits in exercise performance and general health [25]. Due to its cost-effectiveness, CrM is widely used as an ergogenic aid for competitive and recreational athletes’ benefits [14,22,25]. Numerous studies have demonstrated CrM supplementation improves exercise performance and recovery, strength, velocity, and power production, and sustains higher volumes in workouts, which can lead to increased muscle mass [14,26,27,28,29,30,31,32,33]. In this context, firefighters can benefit from enhanced physical capacities and capabilities (e.g., muscular endurance, anaerobic capacity, and improved recovery). The following describes the potential benefits of CrM supplementation in firefighters with reference to how CrM supplementation may also benefit police and military who perform similar occupational tasks.

## 4. Potential Benefits to the Firefighter and Tactical Occupation Community

In the last century, the United States (U.S.) Fire Service has evolved from solely fighting fires to providing all-hazard responses. In addition to structural firefighting, typical fire agencies may offer wildland firefighting, motor vehicle extrication, emergency medical services (EMSs), community-based medicine, water rescue and recovery, hazardous materials mitigation, fire cause and origin investigation, and technical rescue. Recently, fire departments have also been front and center in response to acts of terrorism [34]. During 2020, more than 360,000 U.S. career firefighters and ≈640,000 volunteer firefighters answered daily calls to protect lives and property [35]. The health and wellness of firefighters remain a constant challenge, wherein the risk of CVD and cancer remains high [36,37].

Firefighters engage in periodic and intermittent physical work while exposed to heat stress, austere environmental conditions, and decision-making tasks that can be the difference between life and death [38]. Structural fire scenes can reach ≈65–93 °C at floor level with low-visibility conditions that hinder communication abilities [39]. Moreover, musculoskeletal injuries (e.g., strains and sprains) are the leading cause of injury overall, and the knees, lower back, and shoulders are primarily impacted. Therefore, muscular strength, power, endurance, and appropriate mobility and stability are important to mitigate injury [2,40]. Further, a desirable body composition appears to be correlated with better health, improved performance on occupational task tests, and decreased risk of injury [41,42,43,44].

**Figure 1:**
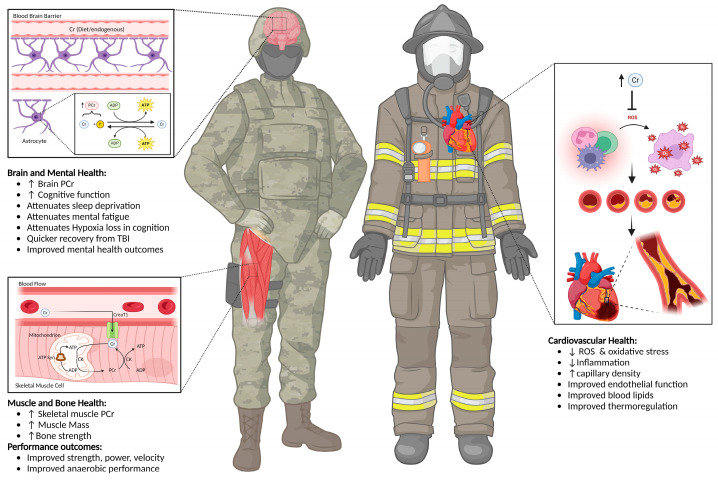
Potential benefits of creatine monohydrate supplementation for firefighters and other related tactical personnel. ADP = adenosine diphosphate, ATP = adenosine triphosphate, CrM = creatine monohydrate, CreaT1 = creatine transporter 1, PCr = phosphocreatine, P = inorganic phosphate, ROS = reactive oxygen species, TBI = traumatic brain injury, ↑ = increase, ↓ = decrease.

Scene awareness and decision-making are also paramount for firefighters [45]. Mental acuity and executive functioning are critical due to the danger of the work. Fire scenes are extremely dangerous, considering at-home products, automobiles, or waste materials that may cause potential exposure to toxic fumes to firefighters. Additionally, firefighters are at risk of minor to major traumatic brain injuries (TBIs), which can impact their sleep and mental health [46]. Considering these occupation-specific factors and the plethora of research on creatine, it is reasonable to assume that this ergogenic aid can favorably impact firefighters and related tactical occupations due to its effects on exercise, cognitive performance, and various aspects of health. Figure 1 displays the potential benefits of CrM for firefighters.

## 5. Creatine and Performance Parameters

Countless studies have shown that CrM supplementation can enhance exercise performance [14,26]. Specifically, CrM supplementation can enhance high-intensity, short-duration exercise performance by enhancing ATP production, which in turn can lead to increased force production, velocity, and power production, improve recovery between sets, increase work output, and enhance training adaptations in the long term [14,26,47,48,49]. Firefighters rely on power production and augment oxidative capacity for efficient on-site performance. Thus, CrM supplementation can enhance aspects of performance and functionality that can translate to better fire-suppressive activity outcomes (e.g., deploying hose lines, forcible entry, carrying heavy equipment) [50] and enhance firefighter performance and functionality.

### 5.1. Short-Term Creatine Monohydrate Supplementation

Short-term CrM supplementation can increase skeletal muscle CrM stores following a loading (20 g/day or 0.3 g/kg lean body mass for 7-day) and maintenance phase (5 g or 0.075 g/kg/day of lean body mass). A 5-day CrM loading protocol has been shown to enhance intramuscular CrM stores while improving leg press one-repetition maximum (1-RM) among healthy males [51]. Short-term CrM use can augment muscular strength and power indices [27,52,53,54]. For example, Kreider and colleagues [55] also assessed 15.75 g/day for four weeks among 25 NCAA Division 1 football players and found increased total body weight (+2.42 kg), lean mass (+2.42 kg), bench press lifting volume (+225 kg), the sum of bench press, squat, and power clean lifting volume (+1558 kg), and total work performed during the first five 6-second sprints. Another study demonstrated that elite youth soccer athletes improved peak and mean power output during a Wingate assessment after 14 days of CrM supplementation (0.03 g/kg/day) [27]. Cox et al. [56] also showed that acute CrM use (6 days of 20 g/day) in elite female soccer players improved repeated sprint and agility performance outcomes. One review supported the acute ergogenic effects of CrM supplementation coupled with resistance training (RT) compared to RT alone, with CrM eliciting an 8% increase in muscular strength [57]. Other reviews have highlighted Cr’s ability to augment lower and upper body strength [26,31,58].

Creatine regulates oxidative phosphorylation by shuttling inorganic phosphate from the mitochondria to the cytosol, maintaining cellular bioenergetics [59,60,61]. However, the current efficacy of creatine supplementation is less clear for endurance exercise [26]. Although evidence currently does not support increases in maximal aerobic capacity (VO_2max_ or VO_2peak_) [26], a few studies have assessed creatine’s effects on endurance performance, with some ergogenic effects noted. Nelson et al. [62] demonstrated that 20 g/day for 7 days of CrM supplementation lowered oxygen consumption and heart rate at the end of the first five graded exercise test stages, as well as improved ventilatory anaerobic threshold (pre-CrM 66% to post-CrM 78%). Graef and colleagues [63] reported that four weeks of creatine citrate (10 g/day, providing 57.7% creatine by molecular weight [22]) supplementation improved the ventilatory anaerobic threshold by 16% (6% higher than placebo). Taken together, there may be some benefit of creatine supplementation (CrM and creatine citrate) on improving ventilatory threshold during an endurance bout of exercise. Moreover, competitive rowers experienced an improvement in 1000 m rowing times after 5 days of 0.25 g/kg/day of CrM ingestion [64]. Similarly, male paddlers increased total work capacity during high-intensity rowing exercises lasting up to five minutes after 5 days of 20 g/day CrM ingestion [65]. Lastly, Miura et al. [66] showed increased anaerobic work capacity in healthy males during cycle ergometry exercise after 5 days of high-dose CrM supplementation.

Some evidence suggests acute CrM use (i.e., 0.3 g/kg for 5 to 7 days) improves fatigue in athletes performing subsequent exercise bouts (e.g., powerlifters and soccer players) [67,68]. Another study showed improved fatigue in repeated Wingate exercises in un young trained males after 6 days of CrM supplementation [69]. The rate of recovery has also been assessed with acute CrM use. For example, Birch et al. [70] also showed enhanced successive cycling performance after acute CrM use, indicating higher sustained power output for repeated cycling bouts. Additionally, CrM has been shown to attenuate the increase in muscle damage biomarkers (e.g., creatine kinase [CK] and lactate dehydrogenase [LDH]) after stressful exercise [71,72,73,74]. A meta-analysis on short- and long-term CrM use noted CrM’s ability to attenuate CK and LDH concentrations after strenuous exercise, with more favorable results seen with acute durations and higher dosages [73]. Currently, more evidence supports short-term higher dosages of CrM (≥20 g/day) for attenuating muscle damage markers compared to longer-term CrM consumption, which may indicate the importance of dose vs. duration concerning CrM and muscle damage [73]. Endurance athletes may also benefit from creatine supplementation as a means of enhancing muscle glycogen loading [75,76,77,78,79], improving interval training and/or the ability to sprint toward the end of endurance events [77], and enhancing thermoregulation during prolonged exercise in the heat [14,32,80,81,82,83,84].

### 5.2. Chronic Supplementation

Chronic CrM supplementation is characterized by ingesting approximately 5 g/day or 0.075 g/kg/day after 5–7 days of CrM loading. As shown in many studies, prolonged CrM supplementation can augment training adaptations by facilitating increased training volume [30,55,85,86,87,88,89]. For example, male powerlifters experienced increased bench press strength, endurance, and body mass after 26 days of CrM supplementation [85]. Creatine monohydrate supplementation has been shown to improve sport-related [87,90,91] and occupational performance among athletes and tactical athletes [92]. For example, in soldiers, a 6-day loading protocol (i.e., 20 g/day) followed by 6 g/day for four weeks of CrM supplementation favorably impacted exercise performance outcomes and thermoregulation in a hot environment [92]. Volek and colleagues [93] have also shown that 12 weeks of CrM supplementation (i.e., 25 g/day for 7 days’ loading followed by 5 g/day for 11 weeks) among healthy resistance-trained men led to increased body mass (6.3%) and fat-free mass (6.3%) compared to placebo. Furthermore, the researchers found increased type I (35%), IIA (36%), and IIAB (35%) muscle fiber cross-sectional areas, suggesting CrM’s ability to augment training adaptations. In addition, CrM has been shown to reduce heart rate, which, combined with improved thermoregulation (reduced body temperatures) [32,80,81,82,83,84,92], can theoretically improve firefighter-specific performance.

The evidence regarding chronic CrM supplementation on endurance performance is less clear. Graef et al. [63] noted improved time to exhaustion and ventilatory threshold with no changes in total work in active males supplementing with CrM for 5 days a week of 10 g/day for 30 days. Kendall et al. [94] found ergogenic effects on critical power, but not on anaerobic work capacity in high-intensity interval training. Furthermore, 3 g/day of CrM supplementation for 28 days improved exercise economy, but not respiratory exchange ratio (RER), blood lactate, or sprint performance at supramaximal speeds in endurance-trained males [95]. Creatine monohydrate also augments recovery after high-intensity bouts of exercise and between intermittent sporting events. For instance, Kreider and colleagues [55] showed that 28 days of CrM supplementation in collegiate football players improved total work completed during multiple successive sprint bouts. The interested reader is directed to the Wax et al. [26] 2021 review, wherein numerous studies demonstrate that acute and chronic (>2 weeks) CrM favorably impact performance and recovery.

Overall, abundant evidence suggests health-conferring benefits and ergogenic effects of short- and long-term CrM supplementation in many populations. It is worth mentioning that there are a few studies that have assessed the impact of creatine supplementation among other tactical personnel [92,96]. First, de Silveira and colleagues [96] demonstrated that 12 weeks of creatine and glutamine supplementation (i.e., creatine and glutamine: 0.3 g/kg/day for 7 days’ loading followed by 0.03 g/kg/day 11-week maintenance) did not lead to greater ergogenic benefits on physical performance among 32 male military police officers when compared to creatine alone, glutamine alone, or a placebo (corn flour). However, Bennett et al. [92] did demonstrate that creatine supplementation (i.e., 20 g/day for 6 days’ loading followed by 6 g/day of maintenance for four weeks) resulted in increased pull-ups performed with no adverse health outcomes. Lastly, Warber et al. [97] demonstrated that CrM supplementation (i.e., providing 24 g of CrM contained within a sports bar; 1 bar per day) for 5 days did not improve military obstacle course performance. To date, only one study has assessed the impact of CrM among firefighters on an occupational performance task [50]. However, while empirical evidence is needed, it is reasonable to hypothesize that CrM can enhance muscular strength and power indices, functionality, and vitality and improve firefighters’ recovery.

## 6. Creatine and Body Composition

Body composition is an important determinant of health in firefighters [36]. Firefighters who are classified as overweight or obese are at a high risk of suffering cardiovascular complications, which would negatively impact their occupational performance and increase the prevalence of duty-related fatality [98,99]. Diet and exercise influence body composition, as the balance between caloric intake and energy expenditure influences weight fluctuations [100]. Beyond body weight, understanding how various interventions impact tissue composition (i.e., muscle and fat mass) is critical from a health and performance perspective [100].

Creatine monohydrate supplementation has been purported to alter body composition [101,102,103]. Research has shown that combining CrM supplementation and exercise (primarily RT) improves aspects of body composition. Specifically, several systematic reviews and meta-analyses [101,103,104,105,106,107] on CrM supplementation have shown greater gains in whole-body lean mass (1.1–1.37 kg) compared to placebo in young and older adults. In addition, Burke et al. [106] performed a systematic review and meta-analysis that included 10 studies and found significant improvements in limb muscle thickness (0.10–0.16 cm) from CrM supplementation. These muscle and lean mass benefits may be related to CrM influencing cellular hydration status, glycogen and calcium kinetics, myogenic regulatory factors, satellite cell proliferation and activation, growth factors, inflammation, oxidative stress, protein catabolism, and/or training volume [93,104].

Regarding fat mass, there is some evidence in animals that CrM metabolism influences whole-body energy expenditure in adipocytes [108,109,110]. For example, in transgenic mice that lacked adipose creatine transporters, higher levels of adiposity and lower levels of whole-body energy expenditure were observed, which suppressed fat oxidation over time [108,109]. However, the effects of CrM supplementation on measures of fat mass and body fat percentage (BF%) in humans are small and likely not clinically relevant. A previous meta-analysis showed that healthy older adults (n = 609; 19 studies; ≥50 years) who supplemented with creatine (≥2 g/day) and performed RT (2–3 times/week for up to 1 year) experienced a significant reduction in BF% (0.55%; CI: −1.08 to −0.03; *p* = 0.04); however, there was no change in fat mass compared to RT alone [102].

Lastly, there is some evidence that CrM combined with RT and walking can enhance the properties of bone health [111,112,113,114]. CrM can alter bone strength through direct and indirect mechanisms [115]. Directly, CrM supplementation has been shown to increase the activity of osteoblasts (bone-forming cells) in cell culture models [116]. Indirectly, CrM can enhance muscle mass and strength, thereby causing more stress on the body. Recently, Chilibeck et al. [117] examined the effects of CrM (0.14 g/kg/day) combined with RT (3 days a week) and walking (6 days a week) on bone over 2 years in 237 postmenopausal females. Results showed that CrM improved bone strength and geometry indices over time.

Collectively, there is evidence that combining CrM supplementation and RT increases whole-body lean mass and regional muscle accretion in young and older adults. Further, CrM appears to produce a minimal yet beneficial effect on body fat percentage in older adults. Finally, CrM has some favorable effects on measures of bone biology in older adults. However, the effects of CrM on body composition in firefighters are unknown and warrant future research.

## 7. Cardiovascular and Antioxidant Impacts

It is well established that firefighters have a high risk of CVD and premature mortality. Occupation-related stressors can lead to an increase in free radical generation and subsequent oxidative stress, which is implicated in CVD risk. Several studies demonstrate that oxidative stress biomarkers increase in response to fire-suppressive activities [6,7,118]. Recent comprehensive reviews have identified nutritional strategies to aid firefighters in combating CVD risk [1,2].

### 7.1. Vascular Health

Creatine monohydrate has garnered recent attention for its potential impact on cardiovascular health [119,120,121]. Only a handful of studies have been completed on the direct effects of CrM supplementation on cardiovascular health. For example, Arciero and colleagues [122] assessed the impact of CRM and RT for 28 days (i.e., 5 × 4 g for 5 days followed by 10 g/day for the remainder of the study) on limb blood flow among 30 healthy, resistance-untrained male subjects. Interestingly, only the CrM and RT groups experienced increases (*p* < 0.05) in calf (30%) and forearm (38%) limb blood flow, while the CrM and RT-only groups did not [122]. Total body water (TBW) also increased (2.2 L and 1.7 L for the creatine-only and CrM and RT groups, respectively) following the 28-day intervention. The authors proposed that the increase in TBW could impact plasma volume, venous return, and cardiac output; however, the mechanism of action for how CrM supplementation can augment limb blood flow remains unknown. The authors concluded that their novel results suggested a synergistic effect of CrM and RT concerning limb blood flow [122].

In another study assessing the impact of CrM on cardiovascular health, Sanchez-Gonzalez et al. [123] administered 10 g/day of CrM or a placebo for 21 days to 16 healthy young males. The participants experienced an attenuated increase in systolic blood pressure at 5 min post-exercise (PLA: 14 ± 2, Cr: 5 ± 2 mmHg) and heart rate responses at 5 min post-exercise (PLA: 28 ± 4, CrM 16 ± 2 bpm) and 15 min post-exercise (PLA: 21 ± 3, Cr: 11 ± 2 bpm) [123]. In addition, the CrM group experienced quicker recovery to resting values for systolic blood pressure and heart rate. Lastly, the CrM group experienced a blunted brachial–ankle pulse wave velocity response to the exercise bout. Collectively, these findings suggest that CrM supplementation can favorably impact hemodynamics and vascular health following acute exercise [122,123]. However, some reports do not support CrM affecting vascular health outcomes (i.e., pulse wave velocity) [124,125]. For example, Aubry et al. [124] assessed the impact of CrM supplementation of 21 g/day for 7 days on central and peripheral pulse wave velocity and found no differences between the CrM and placebo groups. While only speculative, a longer supplementation duration is likely needed.

Two other investigations regarding creatine and vascular health, particularly the impact of CrM supplementation on microvasculature, are available. First, Moraes et al. [126] assessed the impact of a loading dose (i.e., 20 g/day) of CrM on microvascular reactivity, microcirculation, and skin capillary density among 40 young, moderately active males. The CrM supplementation resulted in reductions in low-density-lipoprotein cholesterol (LDL-C) and total cholesterol (TC), as well as increased skin functional capillary density and capillary recruitment post-occlusive reactive hyperemia (i.e., indication of reperfusion after brief ischemia). Van Bavel et al. [127] assessed the impact of 5 g/day of CrM for 3 weeks among 49 vegan subjects. Interestingly, the subjects experienced reduced homocysteine levels (*p* = 0.0282; 13%), as well as reductions in LDL-C (*p* = 0.110; 7%) and TC (*p* = 0.078; 3%). Capillary density also increased within the CrM group compared to the placebo group. While the exact mechanism remains unclear, these data suggest that CrM may positively impact microvascular and cardiovascular health. Furthermore, considering the improvements noted by Moraes et al. [126] and Van Bavel et al. [127], it is worth noting that CrM supplementation has been shown to improve blood lipid profiles. Kreider et al. [55] demonstrated that 28 days of CrM supplementation (15.75 g/day) resulted in higher HDL and lower VLDL among NCAA Division 1 athletes engaged in resistance and agility–sprint training compared to the placebo group. In addition, Earnest and colleagues [128] demonstrated that 56 days of CrM supplementation (5 g/day) led to reductions in triglycerides and VLDL after 4, 8, and 12 weeks compared to baseline values (−23%, −22%, and −26%, respectively).

### 7.2. Inflammation and Oxidative Stress

There are several potential ways in which CrM supplementation may confer a benefit to cardiovascular health. In particular, CrM has been shown to reduce damage from free radicals and reactive oxygen species (ROS), lessen mitochondria-specific ROS [129], attenuate inflammation, and reduce circulating homocysteine concentrations [127,130], which highlights the potential mechanisms by which creatine can impact cardiovascular health.

There has been an increase in interest regarding Cr’s antioxidant potential and the subsequent health and physical performance benefits that may accompany the attenuation of OS. The mechanism by which CrM acts as an antioxidant is not fully understood; however, CrM has been shown to increase antioxidant enzyme activity and capacity to neutralize ROS [72,131,132]. The muscle is an important site of free radical and ROS generation, and approximately 90% of the body’s CrM stores are within skeletal muscle. Therefore, CrM is primarily located within the ideal position to serve as an antioxidant.

To date, only a handful of studies have directly assessed CrM supplementation and OS in humans. Kingsley et al. [133] assessed the impact of a 7-day loading dose of CrM (20 g) among 18 active males who completed exhaustive incremental cycling trials. The CrM treatment did not favorably impact markers of lipid peroxidation or plasma concentrations of non-enzymatic antioxidants. Rahimi [134] assessed the impact of CrM supplementation on the OS response and DNA oxidation following a bout of RT. Following a 20 g/day for a 7-day supplementation protocol, the CrM group experienced a reduction in urinary 8-hydroxy-2-dayeoxyguanosine (8-OGdG) and plasma malondialdehyde (MDA) responses to RT. A recent study by Amiri and Sheikholeslami-Vatani [135] demonstrated that a 10-week RT program coupled with CrM (0.1 g/kg of body mass) resulted in higher GPX enzyme levels, denoting a synergistic effect of creatine. Interestingly, two other reports do not support CrM’s ability to serve as an antioxidant, with one report that may even indicate a pro-oxidant effect [136]. The literature regarding creatine supplementation and antioxidant effects is equivocal, and further work is needed to better understand how and if CrM supplementation can be leveraged as a direct or indirect antioxidant.

In terms of impact on inflammation, there are data to suggest that CrM supplementation may serve as an anti-inflammatory agent that acts on certain immune cells, such as Toll-like receptors (TLRs), activated macrophages, T cells, and the cytokines that are released or activate these cells [137,138,139,140]. Research suggests CrM can cause reductions in pro-inflammatory cytokines, such as tumor necrosis factor α (TNF-α) and prostaglandin E2 (PGE2), eliciting decreased inflammation post-exercise [141]. Santos and colleagues [141] assessed the impact of CrM supplementation (20 g/day for 5 days) among runners completing a 30 km race. The CrM group experienced attenuated changes in PGE2 (60.9%) and TNF-α (33.7%), while the placebo group experienced increases in PGE2 (6.6-fold) and TNF-α (2.34-fold). Bassit et al. [142] assessed the impact of 20 g/day for 5 days of CrM supplementation before an Ironman completion and found blunted increases in IL-1β, IL-6, INF-α, and TNF-α. Rawson et al. [143] assessed the impact of CrM supplementation (0.3 g/kg of body mass) among 23 healthy weight-trained males and found no differences in the inflammatory response to exercise (i.e., C-reactive protein [CRP]), which was used as part of the assessment of recovery to exercise. Another study by Oliverica et al. [144] found no effects of CrM supplementation of 5 g/day for 12 weeks coupled with RT on the inflammatory cytokines IL-6, IL-10, CRP, or monocyte chemoattractant protein 1. Taken together, the present body of literature demonstrates a mixed effect of CrM supplementation on markers of inflammation.

There are mixed results for the effects of CrM supplementation on markers of OS and inflammation, and more research is needed to understand better the exact mechanism of how CrM can lessen both OS and inflammation. In addition, post-exercise reduction in OS and inflammation may be important in recovery from exhaustive exercise among firefighters. While only speculative, the firefighter may benefit from supplementation with CrM regarding enhanced recovery, lessened OS and inflammatory response to exercise, and reduced basal biomarkers of OS and inflammation related to CVD risk.

## 8. Creatine Supplementation and Thermoregulation

### 8.1. Physiological Responses and Thermoregulatory Strain

Firefighters are exposed to unique and challenging environmental conditions, further heightening the already challenging physical demands of the occupation [145,146,147]. Moreover, these physiological demands are exacerbated when wearing personal protective equipment and self-contained breathing apparatus, adding ≈20–45 kg of weight and ultimately leading to decrements in work capacity [148,149,150]. Specifically, the heat and ambient temperatures that firefighters are exposed to during fire-suppression activities underpin the driving mechanism contributing to thermoregulatory stressors and resulting physiological burdens. During firefighter activities, radiant heat fluxes between 5 and 10 kWm^−2^ have been reported, with extreme cases resulting in up to 42 kWm^−2^ [151]. This can subsequently influence ambient temperatures during fire-suppression activities, exceeding 100 °C, increasing core temperature above 38 °C [5,146]. As a result, these environmental conditions and the resulting core temperatures experienced by firefighters often result in notable fluid loss. For example, during 3 h of live fire training exercises, it was reported that substantial body weight loss occurred, primarily from fluid loss in the firefighters participating in the training. Specifically, the firefighters completed 3–4 evolutions of 15–25 min periods, separated by 10–15 min. During that time, firefighters established a positive water supply, advanced hoses, completed forcible entries, extinguished fires, completed forcible entries, search and rescue, and ventilation tasks, which led to losses of 1.9 ± 0.9 kg (2.2% BW loss) [146]. Similarly, during a series of firefighter tasks while inside a training structure containing several live fires, significant increases in hematocrit were observed (pre 43.7 ± 3.1%, post 46.1 ± 2.3%) in firefighters, with concomitant decreases in stroke volume also occurring [3]. As expected, the degree of heat stress imposed on firefighters is elevated when completing fire-suppression activities in hotter conditions. Smith et al. [152] reported greater increases in heart rate, tympanic temperature, blood lactate, ratings of perceived exertion, and state anxiety when completing firefighting activities (while wearing standard firefighting turnout gear) in hot (89.6 °C) conditions compared to neutral (13.7 °C) conditions. Overall, the thermoregulatory strain and elevated fluid loss rates contribute to increasing levels of dehydration, ratings of perceived exertion, and reductions in physical work capacity. Dehydration can also increase heart rate and cardiovascular strain during physical activity, which may subsequently increase risks for cardiac events. As such, any cooling strategy or intervention that can reduce thermoregulatory strain for this population or better maintain hydration levels would mitigate the overall physiological burden during firefighter activities in the heat and reduce risk factors for cardiac events [153].

### 8.2. Creatine Supplementation

Creatine monohydrate has been proposed as a potential dietary supplement of interest for firefighters, as it has been purported to support thermoregulation during exercise in the heat, which would have applications for firefighters during fire-suppression activities. Previous work has found that CrM supplementation can increase TBW, particularly intracellular fluid volume [32,80,81,82,83,84,154,155]. In turn, this may help maintain plasma volume during exercise and improve heat dissipation for better maintenance of core temperature and fluid levels during fire-suppression activities. However, this has yet to be examined in firefighters. There were initial concerns that CrM supplementation may increase the risk of dehydration and cramping during exercise, particularly in hot and humid environments [156]. It was postulated that CrM supplementation may lead to an acute reduction in extracellular fluid volume, as CrM supplementation leads to an osmotic effect as intramuscular CrM content increases, increasing intracellular fluid volumes [28,156]. Based on case reports and anecdotal findings, there were additional concerns that CrM supplementation may also harm the kidneys [156]. However, these concerns have repeatedly been refuted in the literature [33,49,83,156], with more recent evidence indicating that CrM supplementation is safe and helps support fluid balance [14,49,82]. Furthermore, a systematic review and meta-analysis [157] concluded that CrM supplementation does not appear to hinder the body’s ability to dissipate heat, nor does it seem to result in higher rates of adverse effects in active individuals. These findings may have critical applications for tactical athletes/professionals and active individuals who work or exercise outdoors in hot and humid conditions. A study in collegiate male football players found lower rates of cramping, heat-related dehydration, and number of missed practices due to injury in those supplementing with CrM compared to non-CrM users over three years in environmental conditions of 8–40 °C (mean 24.7 ± 9 °C) and 19–98% relative humidity (49.3 ± 17%) [33]. Additional research has found that CrM supplementation does not impair thermal regulation during exercise in the heat or adversely impact fluid balance, core temperature, or incidence of adverse effects following supplementation. For example, following 5 days of CrM supplementation (20 g/day), it was found that trained male cyclists had better maintenance of plasma volume during exercise in the heat; however, this did not translate to improvements in core temperature, hemodynamic responses, ratings of perceived exertion, or cortisol and aldosterone concentrations throughout the exercise trial [158]. Weiss et al. [82] observed significant increases in all body water volumes (total body water, intracellular water, and extracellular water) following 5 days of CrM supplementation (25 g/day). However, the observed increases in body water did not influence changes in core temperature or sweat loss during a 60 min bout of exercise in a temperature-controlled room (37 °C). Similarly, Kilduff et al. [32] reported significant increases in intracellular water and reduced thermoregulatory and cardiovascular responses following 7 days of CrM supplementation (20 g/day) during two constant-load exercise tests to exhaustion in the heat (30.3 °C); however, time to exhaustion was not improved. The increase in total body water has been shown to occur in as little as 3 days following initiation of CrM supplementation [84]. Wright [81] found that 6 days of CrM loading (20 g/day) did not provide any protective thermoregulatory responses during intermittent sprint exercise in the heat (35 °C and 60% relative humidity); however, sprint exercise performance in the heat was significantly improved following CrM supplementation compared to placebo. In a similar study, Volek et al. [154] reported no differences in core temperature following CrM supplementation during exercise in the heat (37 °C) either. However, there was a significant increase in body weight following CrM supplementation, a common occurrence following CrM supplementation [14]. Null findings have similarly been reported in both males and females following CrM supplementation (7 days at 20 g/day) in temperature-controlled conditions (24.05 ± 1.63 °C, relative humidity = 33.28 ± 17.21%) [84].

The addition of other ingredients to CrM supplementation strategies has been purported to improve CrM retention, fluid balance, and physical capacity; however, findings in the literature are mixed. Mendel et al. [83] found similar responses in core temperature and heart rate following CrM + sports drink supplementation during 40 min of exercise in the heat (39 °C) compared to a sports drink alone. Following a 28-day supplementation period, college-aged males experienced a greater increase in total body water following the consumption of CrM (10 g/day) + carbohydrate (68 g/day) compared to carbohydrate alone (68 g/day) [155]. The increase in TBW led to an attenuation in core temperature during a 60 min exercise bout in the heat (37 °C and 25% relative humidity), with no significant differences between CrM and placebo observed for changes in body fat, hematocrit, or heart rate responses during exercise. Easton et al. [159] found that the addition of glycerol to CrM supplementation (0.87 ± 0.21 L) over a 7-day period led to greater increases in TBW compared to creatine alone (0.63 ± 0.33 L) and glycerol + placebo (0.50 ± 0.28 L), yet this increase in TBW did not contribute to improved heart rate, core temperature, or ratings of perceived exertion during an exercise trial in the heat (30 °C and 70% relative humidity). For the CrM + placebo and CrM + glycerol conditions, heart rate, core temperature, and perceived effort were attenuated during exercise in the heat, with no influence on performance.

CrM may also play a role in fluid balance and physical performance following periods of dehydration in active individuals. For example, in a crossover design [160], male participants were initially dehydrated by completing a protocol consisting of standing (20 min), walking (30 min), and cycling (30 min), corresponding to ≈2% body weight loss through dehydration. Participants then completed an 80 min exercise bout, consisting of 20 min sequences (4 min of standing, 13 min of walking, and 3 min of sprinting), repeated four times in an environmental chamber at 33.5 °C and 41% relative humidity [160]. Following the supplementation period (7 days at 21 g/day), participants experienced increased body mass and were better able to maintain plasma volume within the early stages of the dehydration phase [160]. The level of dehydration, changes in sodium and potassium levels, and incidence of cramping were similar across both conditions. Further, following the CrM supplementation period, participants did not experience improvements in peak aerobic capacity or changes in body temperature and physiological strain index during exercise in the heat. Throughout 5 days of intentional body mass reduction (≈3.5% loss), participants supplemented with CrM experienced better maintenance of body mass, isokinetic peak torque, and work at peak torque compared to those who ingested a placebo [161]. However, the CrM supplementation group did experience a reduction in maximal work during a 15-second maximal effort test. In a follow-up study, following a period of rapid body mass reduction (≈4.5% body mass) in well-trained wrestlers, the addition of CrM + glucose to a controlled diet led to more favorable increases in maximal work intensity during a 5 min intermittent intensity cycling test compared to glucose alone throughout a 17 h recovery period [161]. There were no differences in body mass regain during the 17 h recovery period between groups, but a strong correlation between whole-body CrM retention and performance improvements in the cycling test was observed.

In summary, CrM supplementation increases body weight, particularly due to fat-free mass and body water increases. The observed increases in body water volume appear to improve the ability to maintain plasma volume during exercise. This has been shown to lead to modest fluid retention of ≈400–800 mL during exercise in the heat. However, these increases do not appear to consistently improve exercise capacity or thermoregulatory responses during exercise in the heat. Despite the lack of ergogenic benefits, CrM supplementation does not seem to impair thermoregulation during exposure to heat during physical activity, and it does not lead to increased rates of adverse effects (i.e., nausea, muscle cramping, gastrointestinal distress, etc.), despite anecdotal reports and long-standing myths. While promising, more work is needed to examine the effects of CrM supplementation on the thermoregulatory response during firefighter-specific activities and how it may influence overall occupational performance for this population, particularly its ability to mitigate fluid loss during fire-suppression activities.

## 9. Creatine and Mental and Brain Health

It is estimated that 26–30% of the US population is affected by mental health disorders annually [17]. Among firefighters, the prevalence of PTSD has been reported to range from 4.2% to 37.4%, whereas prevalence rates of depression range from 11% to 40% [162]. There is research that indicates that even just a single response to a traumatic event can result in PTSD and/or depression among firefighters [163,164]. In fact, some data suggest up to 90% of firefighters may experience at least one traumatic event throughout their career [165], such as witnessing mass fatalities or child fatalities. Moreover, Stanley and colleagues [166] noted the prevalence of suicidal ideation (47%), making a suicide plan (19%), attempted suicide (16%), and non-suicidal self-harm/injury (16%) among 1027 current and retired firefighters. In addition, another study by Stanley et al. [167] noted that firefighters who responded to a completed suicide were twice as likely to attempt suicide compared to those who did not encounter this type of response. The intersection between nutrition and mental health has garnered increasing attention in recent years, prompting researchers to investigate the potential therapeutic effects of various dietary supplements on cognitive function and psychological well-being. CrM has gained interest for its role in brain health. Forbes et al. [17] recently reviewed the literature on CrM and mental health outcomes, shedding light on the potential mechanisms and implications for brain health.

### 9.1. Creatine and Its Mechanisms of Action

Preliminary evidence suggests that CrM plays a critical, yet underappreciated role in brain function [168]. In the brain, CrM helps maintain cellular energy homeostasis and supports neurotransmission by producing ATP [169,170]. Additionally, CrM possesses antioxidant properties that may confer neuroprotection against OS. A notable example of this is the severe neurological symptoms and developmental delays displayed by children with inborn errors of metabolism, such as CrM synthesis disorders or CrM transporter deficiencies [171,172].

### 9.2. Cognitive Function and Creatine

Numerous studies have explored the effects of CrM on cognitive performance, focusing on tasks demanding short-term memory, attention, and reasoning abilities. Research suggests that CrM supplementation may positively influence cognitive function, particularly in situations involving mental fatigue or sleep deprivation [173,174]. CrM supplementation reduces the decline in cognitive performance (e.g., attention/vigilance) across a period of sleep loss (compared to placebo) [174]. Further, Rae et al. [173] found enhanced working memory and intelligence test scores in vegetarians following CrM supplementation. These findings suggest a potential role for CrM in mitigating cognitive decline commonly associated with fatigue and stress, which has implications for firefighters.

### 9.3. Mental Health and Firefighters

The physically demanding and hazardous nature of firefighting exposes individuals to unique stressors that can have significant implications for their mental health. Firefighters encounter a range of stressors that set their profession apart from other occupations, which includes exposure to traumatic events, such as witnessing death and destruction. Firefighters are also victims of moral injury—profoundly desiring to help in an emergency, but being unable to do so. Subsequently, exposure to these events may result in an elevated risk of developing mental health issues, including PTSD, depression, anxiety, and substance abuse [175,176,177,178]. The constant uncertainty, irregular schedules, lack of sleep, and the job’s physical demands further compound these stressors, potentially leading to long-term psychological consequences [179].

Several factors have been shown to influence firefighters’ mental well-being. Gender disparities reveal that female firefighters often have higher rates of suicidal thoughts and behaviors compared to their male counterparts [180]. Social support, both within and outside the firefighting community, has been identified as a needed resource and preventative measure for the development of mental health disorders [162]. Organizational culture and leadership play a pivotal role in creating a conducive environment for mental health [181]. Conversely, negative workplace culture, lack of peer support, and inadequate training in coping strategies can exacerbate mental health challenges [162,179,180].

Various interventions and support mechanisms have been developed and studied to attempt to address the mental health concerns in this population. Critical incident stress debriefing (CISD) and psychological first aid (PFA) are early interventions aimed at reducing the impact of traumatic events [182]. However, the effectiveness of these interventions remains debated, with concerns raised about potential harm in some instances. Peer support programs, such as the “peer support team” model, have gained traction due to their ability to provide firefighters with a safe space to discuss their experiences [162]. Additionally, proactive strategies like resilience training and mental health education are being integrated into firefighter training curricula to equip individuals with coping skills [183].

The literature on mental health and firefighters underscores the pressing need to prioritize the psychological well-being of these brave professionals. The unique stressors that firefighters face can lead to serious mental health challenges, impacting not only their personal lives but also their ability to carry out their duties effectively. A multifaceted approach is required to mitigate these challenges, encompassing organizational changes, targeted interventions, and a shift in cultural attitudes toward mental health. Fire departments and agencies must collaborate to create supportive environments that encourage open conversations about mental health and ensure that appropriate resources are readily available for those in need. Furthermore, ongoing research is essential to refine existing interventions, identify emerging challenges, and develop new strategies that effectively address the complex interplay between mental health and the demands of firefighting. Understanding and addressing the mental health of firefighters is a crucial endeavor, essential for both the individuals who dedicate their lives to public safety and the communities they serve [179]. More work is needed to identify effective strategies to reduce the adverse effects of psychological stressors incurred by firefighters.

### 9.4. Mood Regulation and Emotional Well-Being

The influence of CrM on mood and emotional well-being has also attracted attention. Some studies have indicated that CrM supplementation might positively affect mood states [169]. For example, Roitman et al. [184] observed significant reductions in depression and fatigue symptoms in individuals with treatment-resistant depression who had been supplementing with Cr. Moreover, a study by Rae et al. [173] demonstrated improved emotional well-being and overall mood in healthy young adults after CrM supplementation. The findings suggest that CrM might regulate neurotransmitters and brain bioenergetics, influencing mood and emotional states [184,185,186]. Alternatively, Gabriel et al. [187] found no effect of CrM supplementation on symptoms of bipolar depression as evaluated by the Montgomery–Åsberg Depression Rating Scale. Similarly, in women, there are inconsistent results on CrM supplementation providing efficacy in the treatment of depression. Kious et al. [188] demonstrated that a combination treatment with CrM and 5-hydroxytryptophan for SSRI- or SNRI-resistant depression was effective. However, Nemets and Levine [189] found that CrM augmentation did not offer any advantage compared with a placebo for symptoms associated with major depression in women, but may still induce a more rapid response in a small subgroup of female patients. CrM did show a potentially promising therapeutic approach for depression in females with a history of methamphetamine use [190].

Little research has been conducted on the effects of CrM supplementation on PTSD. Two studies have described reduced CrM levels in the hippocampal region of the brain [17]. Other nutrients, such as vitamins B6 and B12, may also be protective against depressive symptoms over time in older adults [191]. Further research on the effects of CrM supplementation and other dietary ingredients on post-traumatic stress disorder (PTSD) is needed, particularly among tactical populations.

### 9.5. Neurological Disorders and Creatine

The potential neuroprotective effects of CrM have led researchers to explore its utility as a treatment option for specific neurological disorders [192]. One area of focus has been on neurodegenerative conditions, such as Parkinson’s disease (PD) and Huntington’s disease (HD). A meta-analysis by Bender et al. [193] indicated that CrM supplementation might positively affect PD patients’ motor symptoms and overall function. Similarly, studies have suggested that CrM supplementation may attenuate disease progression and improve motor function in HD patients [194,195]. These findings underscore the neuroprotective potential of CrM and its significance in managing neurodegenerative disorders. Further work is needed to elucidate the potential therapeutic effects for these patients.

### 9.6. Mechanisms Underlying the Creatine–Mental Health Connection

The mechanisms underlying the potential effects of CrM on mental health are complex and multifaceted. The energy-enhancing properties of CrM are vital for maintaining optimal brain function, particularly during periods of increased cognitive demand and stress. Furthermore, Cr’s role in buffering cellular energy levels and supporting neurotransmission underscores its potential influence on cognitive performance and mood regulation. The antioxidant properties of CrM also contribute to its neuroprotective effects, mitigating OS and inflammation, which are implicated in various mental health disorders. However, prospective clinical trials in humans to confirm these findings are currently lacking. CrM has been extensively studied in animals as it pertains to mental disorders, with current research reporting improvements in outcomes following CrM supplementation in rats [17,168,196]. Pazini et al. [197] found in animal models that CrM shares similar effects to ketamine and could potentially serve as a fast-acting antidepressant. The combination of exercise and CrM supplementation had a synergistic effect and was more effective than each treatment option alone in mice with chronic mild stress-induced depression [198].

The literature examining the relationship between CrM supplementation and mental health outcomes presents intriguing evidence of the potential therapeutic benefits of this dietary supplement. While more research is needed to establish definitive causal links and elucidate underlying mechanisms, the existing studies suggest that CrM may offer cognitive enhancement, mood regulation, and neuroprotective effects [17]. The positive impact of CrM on cognitive function and emotional well-being has implications for individuals facing mental fatigue, stress, and neurodegenerative disorders, such as is the case with several tactical populations. Nonetheless, it is essential to approach these findings with caution, recognizing the complexity of the brain’s functioning and the multifactorial nature of mental health [170]. Future research should further explore the mechanisms involved, the potential long-term effects, and the optimal dosing strategies for CrM supplementation and its role in mental health for tactical populations. Ultimately, the emerging insights from this body of literature offer a promising avenue for enhancing mental health through nutritional interventions, providing preliminary evidence for novel therapeutic approaches in mental health care.

### 9.7. Creatine for Concussion and Mild Traumatic Brain Injury

Recently, CrM supplementation has garnered attention for its ability to aid in recovery from mild traumatic brain injury (mTBI), especially among the military, with congressional efforts to improve soldier readiness and reduce the severity of mTBI and concussions among tactical personnel [199]. CrM can cross the blood–brain barrier (BBB) [17], where it plays an essential role in cellular metabolism. Microvascular endothelial cells of the BBB express a Na^+^–Cl^−^-dependent CrM transporter that permits uptake into the brain [200]. However, CrM has low permeability into the BBB, which may necessitate higher consumptions of CrM daily with as high as 30 g/day ingested over a longer time frame to maximize CrM concentrations in the brain compared to skeletal muscle [17]. Moreover, longer consumption of higher amounts of CrM is likely needed to promote improvements in brain bioenergetics, particularly following an mTBI. Briefly, CrM aids in the rapid regeneration of ATP, which ultimately helps reduce the generalized energy crisis that occurs after suffering an mTBI [201,202,203]. However, guanidinoacetic acid (GAA) may better promote increased CrM synthesis in the brain, as it bypasses the BBB more easily [204]. GAA is a precursor to Cr, and its ingestion has been suggested to increase the concentration of CrM in the brain and skeletal muscle [205]. Regardless, CrM is imperative for enhancing brain bioenergetics, e.g., in populations that suffer from cerebrovascular disorders that yield significant amounts of OS in the brain. Two randomized prospective studies in humans have examined the efficacy of CrM supplementation in children after sustaining a moderate-to-severe TBI [206,207]. These open-label studies demonstrated that children supplemented with CrM had significantly improved cognition, communication, self-care, personality, and behavior and considerably reduced occurrences of headaches, dizziness, and fatigue compared to the control group [208]. Based on these preliminary studies, CrM shows promise for the management of TBI symptoms under sleep loss and physiological and psychosocial stress post-acute phase TBI. However, given that these studies were evaluating patients with an mTBI for an extended recovery period after post-acute phase TBI, it is not known if CrM would improve outcomes in individuals whose symptoms resolved and returned to duty/play immediately post-acute phase TBI (<2 weeks).

## 10. Creatine and Sleep Deprivation

Fatigue from sleep deprivation among firefighters is likely to increase the risk of injury, operational failure, or the odds of making a fatal mistake on duty [209,210,211]. Largely, this is because sleep is a restorative process for the brain that clears metabolic waste (e.g., free radicals) and allows nerve cells to restore cellular energy (i.e., ATP) [212]. In addition, sleep deprivation depletes ATP stores [213], which can subsequently impact cellular signaling and vigilance [214]. Given its metabolic role in aiding ATP restoration [215], CrM has been considered for use in alleviating some of the adverse effects of sleep deprivation.

Supplemented CrM is known to be able to cross the BBB [216] and has been demonstrated to improve central executive task performance during sleep deprivation [217] and when in conjunction with moderate-intensity exercise when the tasks were sufficiently demanding [174]. Upon further examining the impact of supplementation strategies on cognitive functions, CrM is most likely to have an effect when cognitive processes are under stress, such as during periods of sleep deprivation, experimental hypoxia, or when engaging in more intricate and demanding tasks [18]. Also, CrM supplementation has been shown to enhance exercise performance and affect cognitive function, neuroprotection, and circadian rhythms [218]. Recently, Gordji-Nejad and colleagues [217] assessed the impact of a single dose of CrM (0.35 g/kg) on cognitive assessment during a state of sleep deprivation. The high single doses of CrM reversed metabolic alterations and fatigue-related cognitive impairments among the study participants. While more research is warranted to build upon these findings, especially among firefighters, these results are important for firefighters who often experience periods of sleep loss and sleep deprivation.

## 11. Safety and Side Effects

Creatine has been classified as generally recognized as safe (GRAS) by the United States Food and Drug Administration (https://www.fda.gov/media/143525/download; accessed on 1 July 2023), and there is substantial evidence to support the safety of creatine use across a wide range of populations. The 2017 International Society of Sports Nutrition comprehensively reviewed the literature surrounding the safety profile of creatine [14]. As such, the only consistent side effect that has been reported following CrM supplementation is weight gain [15,48,219,220,221,222]. Studies examining the validity of purported side effects have shown that CrM either does not cause the purported side effects anecdotally reported (e.g., gastrointestinal distress, muscle cramping, etc.) or may reduce the incidence of these side effects [49]. In fact, minimal to no adverse side effects are noted based upon the available data on short- and long-term supplementation ranging from 0.3 to 0.8 g/kg/day of CrM up to 5 years within a wide range of study populations (e.g., infants, elderly, and athletes) [14,25,49,223]. An exhaustive literature review is outside the scope of this narrative review. Therefore, the interested reader is encouraged to review the common questions and misconceptions regarding creatine use [49], as several concerns have been reviewed, and the available data to date suggest that CrM supplementation in the recommended dosages is safe for a wide range of health and clinical populations. For instance, Gualano et al. [224] assessed the impact of 5 g/day for 12 weeks of CrM supplementation among older adults with type 2 diabetes who were at higher risk of developing kidney disease, and found no signs of deterioration of kidney function. Overall, the research on creatine is compelling, as the experimental and controlled research indicates that CrM supplementation is safe for both healthy and clinical populations. There are data that also suggest that dietary creatine is associated with a reduced risk of cancer [225], reproductive issues [226], and depression [186], with no associations with kidney dysfunction [227,228]. It is worth noting that it is always advisable for those starting a new nutritional regimen or supplementation practice to consult with their physician if they have any concerns regarding the safety of the new practice.

## 12. Future Directions

This narrative review highlights physical, occupational, and health-related domains wherein CrM supplementation can aid firefighters and other tactical personnel in readiness for their stressful encounters. While this narrative, scoping review provides a gross overview of the benefits of CrM use among firefighters, future efforts should employ a systematic review of the literature utilizing guidelines defined by PRISMA 2020 to enhance the transparency and quality of the conclusions. There is a plethora of evidence to support the use of CrM supplementation among firefighters; however, to date, only one study has been completed regarding CrM supplementation among firefighters. Therefore, more research is warranted to assess the impact of this key nutrient directly within the context of firefighting (as well as among other tactical personnel). In particular, future studies should aim to assess the effects of CrM’s ability to (1) improve occupation-specific performance on firefighting tasks and operations (i.e., simulated fire ground testing and live fire training evolution), (2) attenuate the stress response to firefighting via its antioxidant/anti-inflammatory potential and thus improve recovery, and (3) improve cognitive, mood, brain, and mental health outcomes among firefighters.

## 13. Conclusions

CrM can benefit firefighters across several domains relevant to firefighting. Future work should emphasize acute and prolonged CrM supplementation studies to assess the impact on occupation-specific performance. There is a plethora of evidence to suggest that CrM can improve exercise performance and augment training adaptations, which can translate to improved physical and occupational abilities during live fire responses. CrM may also offer a health benefit via its antioxidant/anti-inflammatory properties, which can improve cardiovascular health and brain health outcomes. Firefighters who are susceptible to brain injury (concussion) and stress (sleep loss) could benefit from CrM supplementation, particularly a higher dose (20–30 g). This area is of particular interest for future research, especially concerning the tactical athlete. Firefighters, police officers, and military personnel who perform similar occupational tasks should consider supplementing their diet with 5 g/day of CrM to enhance exercise and occupational performance outcomes, whereas high doses (20–30 g/day) are likely needed to confer mental and brain health benefits.

## Data Availability

Not applicable.

## References

[1] Gonzalez D.E., Lanham S.N., Martin S.E., Cleveland R.E., Wilson T.E., Langford E.L., Abel M.G. (2024). Firefighter Health: A Narrative Review of Occupational Threats and Countermeasures. Healthcare.

[2] Gonzalez D.E., McAllister M.J., Waldman H.S., Ferrando A.A., Joyce J., Barringer N.D., Dawes J.J., Kieffer A.J., Harvey T., Kerksick C.M. (2022). International society of sports nutrition position stand: Tactical athlete nutrition. J. Int. Soc. Sports Nutr..

[3] Smith D.L., Manning T., Petruzzello S.J. (2001). Effect of strenuous live-fire drills on cardiovascular and psychological responses of recruit firefighters. Ergonomics.

[4] Larsen B., Snow R., Williams-Bell M., Aisbett B. (2015). Simulated Firefighting Task Performance and Physiology under Very Hot Conditions. Front. Physiol..

[5] Angerer P., Kadlez-Gebhardt S., Delius M., Raluca P., Nowak D. (2008). Comparison of cardiocirculatory and thermal strain of male firefighters during fire suppression to exercise stress test and aerobic exercise testing. Am. J. Cardiol..

[6] McAllister M.J., Gonzalez A.E., Waldman H.S. (2022). Impact of time restricted feeding on markers of cardiometabolic health and oxidative stress in resistance-trained firefighters. J. Strength Cond. Res..

[7] Gonzalez D.E., Waldman H.S., McAllister M.J. (2023). The metabolic and physiological demands of a simulated fire ground test versus a live-fire training evolution in professional firefighters. Int. J. Exerc. Sci..

[8] Gonzalez D.E., Dillard C.C., Johnson S.E., Martin S.E., McAllister M.J. (2023). Physiological stress responses to a live-fire training evolution in career structural firefighters. J. Occup. Environ. Med..

[9] Kales S.N., Smith D.L. (2017). Firefighting and the Heart: Implications for Prevention. Circulation.

[10] Hunter A.L., Shah A.S., Langrish J.P., Raftis J.B., Lucking A.J., Brittan M., Venkatasubramanian S., Stables C.L., Stelzle D., Marshall J. (2017). Fire simulation and cardiovascular health in firefighters. Circulation.

[11] Huang C.-J., Webb H.E., Zourdos M.C., Acevedo E.O. (2013). Cardiovascular reactivity, stress, and physical activity. Front. Physiol..

[12] Janczura M., Rosa R., Dropinski J., Gielicz A., Stanisz A., Kotula-Horowitz K., Domagala T. (2021). The Associations of Perceived and Oxidative Stress with Hypertension in a Cohort of Police Officers. Diabetes Metab. Syndr. Obes..

[13] Sullivan-Kwantes W., Cramer M., Bouak F., Goodman L., Sookermany A.M. (2020). Environmental Stress in Military Settings. Handbook of Military Sciences.

[14] Kreider R.B., Kalman D.S., Antonio J., Ziegenfuss T.N., Wildman R., Collins R., Candow D.G., Kleiner S.M., Almada A.L., Lopez H.L. (2017). International Society of Sports Nutrition position stand: Safety and efficacy of creatine supplementation in exercise, sport, and medicine. J. Int. Soc. Sports Nutr..

[15] Kerksick C.M., Wilborn C.D., Roberts M.D., Smith-Ryan A., Kleiner S.M., Jager R., Collins R., Cooke M., Davis J.N., Galvan E. (2018). ISSN exercise & sports nutrition review update: Research & recommendations. J. Int. Soc. Sports Nutr..

[16] Candow D.G., Forbes S.C., Ostojic S.M., Prokopidis K., Stock M.S., Harmon K.K., Faulkner P. (2023). “Heads Up” for Creatine Supplementation and its Potential Applications for Brain Health and Function. Sports Med..

[17] Forbes S.C., Cordingley D.M., Cornish S.M., Gualano B., Roschel H., Ostojic S.M., Rawson E.S., Roy B.D., Prokopidis K., Giannos P. (2022). Effects of Creatine Supplementation on Brain Function and Health. Nutrients.

[18] Dolan E., Gualano B., Rawson E.S. (2019). Beyond muscle: The effects of creatine supplementation on brain creatine, cognitive processing, and traumatic brain injury. Eur. J. Sport. Sci..

[19] Roschel H., Gualano B., Ostojic S.M., Rawson E.S. (2021). Creatine Supplementation and Brain Health. Nutrients.

[20] Havenetidis K. (2016). The use of creatine supplements in the military. J. R. Army Med. Corps.

[21] Sheppard H.L., Raichada S.M., Kouri K.M., Stenson-Bar-Maor L., Branch J.D. (2000). Use of creatine and other supplements by members of civilian and military health clubs: A cross-sectional survey. Int. J. Sport. Nutr. Exerc. Metab..

[22] Kreider R.B., Jäger R., Purpura M. (2022). Bioavailability, Efficacy, Safety, and Regulatory Status of Creatine and Related Compounds: A Critical Review. Nutrients.

[23] Farshidfar F., Pinder M.A., Myrie S.B. (2017). Creatine Supplementation and Skeletal Muscle Metabolism for Building Muscle Mass—Review of the Potential Mechanisms of Action. Curr. Protein Pept. Sci..

[24] Jager R., Purpura M., Shao A., Inoue T., Kreider R.B. (2011). Analysis of the efficacy, safety, and regulatory status of novel forms of creatine. Amino Acids.

[25] Kreider R.B., Stout J.R. (2021). Creatine in Health and Disease. Nutrients.

[26] Wax B., Kerksick C.M., Jagim A.R., Mayo J.J., Lyons B.C., Kreider R.B. (2021). Creatine for Exercise and Sports Performance, with Recovery Considerations for Healthy Populations. Nutrients.

[27] Yáñez-Silva A., Buzzachera C.F., Piçarro I.D.C., Januario R.S., Ferreira L.H., McAnulty S.R., Utter A.C., Souza-Junior T.P. (2017). Effect of low dose, short-term creatine supplementation on muscle power output in elite youth soccer players. J. Int. Soc. Sports Nutr..

[28] Williams M.H., Branch J.D. (1998). Creatine supplementation and exercise performance: An update. J. Am. Coll. Nutr..

[29] Volek J.S., Kraemer W.J., Bush J.A., Boetes M., Incledon T., Clark K.L., Lynch J.M. (1997). Creatine supplementation enhances muscular performance during high-intensity resistance exercise. J. Am. Diet. Assoc..

[30] Vandenberghe K., Goris M., Van Hecke P., Van Leemputte M., Vangerven L., Hespel P. (1997). Long-term creatine intake is beneficial to muscle performance during resistance training. J. Appl. Physiol..

[31] Lanhers C., Pereira B., Naughton G., Trousselard M., Lesage F.X., Dutheil F. (2015). Creatine Supplementation and Lower Limb Strength Performance: A Systematic Review and Meta-Analyses. Sports Med..

[32] Kilduff L.P., Georgiades E., James N., Minnion R., Mitchell M., Kingsmore D., Hadjicharalambous M., Pitsiladis Y. (2004). The effects of creatine supplementation on cardiovascular, metabolic, and thermoregulatory responses during exercise in the heat in endurance-trained humans. Int. J. Sport. Nutr. Exerc. Metab..

[33] Greenwood M., Kreider R.B., Melton C., Rasmussen C., Lancaster S., Cantler E., Milnor P., Almada A. (2003). Creatine supplementation during college football training does not increase the incidence of cramping or injury. Mol. Cell. Biochem..

[34] Byrnes A. (2023). Firefighters Protecting a Nation: Historical Perspectives and a Modern, All-Hazards Approach. The Distributed Functions of Emergency Management and Homeland Security.

[35] Fahy R.F., Evarts B., Stein G.P. (2022). US Fire Department Profile 2020.

[36] Lockie R.G., Dulla J.M., Higuera D., Ross K.A., Orr R.M., Dawes J.J., Ruvalcaba T.J. (2022). Body Composition and Fitness Characteristics of Firefighters Participating in a Health and Wellness Program: Relationships and Descriptive Data. Int. J. Environ. Res. Public Health.

[37] Soteriades E.S., Kim J., Christophi C.A., Kales S.N. (2019). Cancer Incidence and Mortality in Firefighters: A State-of-the-Art Review and Meta-َAnalysis. Asian Pac. J. Cancer Prev..

[38] Smith D.L., Barr D.A., Kales S.N. (2013). Extreme sacrifice: Sudden cardiac death in the US Fire Service. Extrem. Physiol. Med..

[39] Willi J.M., Horn G.P., Madrzykowski D. (2016). Characterizing a firefighter’s immediate thermal environment in live-fire training scenarios. Fire Technol..

[40] Abel M.G., Palmer T.G., Trubee N. (2015). Exercise program design for structural firefighters. Strength. Cond. J..

[41] Nogueira E.C., Porto L.G.G., Nogueira R.M., Martins W.R., Fonseca R.M., Lunardi C.C., de Oliveira R.J. (2016). Body composition is strongly associated with cardiorespiratory fitness in a large Brazilian military firefighter cohort: The Brazilian firefighters study. J. Strength. Cond. Res..

[42] Bond C.W., Waletzko S.P., Reed V., Glasner E., Noonan B.C. (2022). Retrospective longitudinal evaluation of male firefighter’s body composition and cardiovascular health. J. Occup. Environ. Med..

[43] Walker A., Beatty H.E.W., Zanetti S., Rattray B. (2017). Improving body composition may reduce the immune and inflammatory responses of firefighters working in the heat. J. Occup. Environ. Med..

[44] Jahnke S., Poston W., Haddock C., Jitnarin N. (2013). Obesity and incident injury among career firefighters in the central United States. Obesity.

[45] Maguire B.J., O’Meara P., O’Neill B.J., Brightwell R. (2018). Violence against emergency medical services personnel: A systematic review of the literature. Am. J. Ind. Med..

[46] Strack J., Torres V., Pennington M., Cardenas M., Dupree J., Meyer E., Dolan S., Kruse M., Synett S., Kimbrel N. (2021). Psychological distress and line-of-duty head injuries in firefighters. Occup. Med..

[47] Cooper R., Naclerio F., Allgrove J., Jimenez A. (2012). Creatine supplementation with specific view to exercise/sports performance: An update. J. Int. Soc. Sports Nutr..

[48] Kreider R.B. (2003). Effects of creatine supplementation on performance and training adaptations. Mol. Cell. Biochem..

[49] Antonio J., Candow D.G., Forbes S.C., Gualano B., Jagim A.R., Kreider R.B., Rawson E.S., Smith-Ryan A.E., VanDusseldorp T.A., Willoughby D.S. (2021). Common questions and misconceptions about creatine supplementation: What does the scientific evidence really show?. J. Int. Soc. Sports Nutr..

[50] Elstad K., Malone C., Luedke J., Jaime S.J., Dobbs W.C., Almonroeder T., Kerksick C.M., Markert A., Jagim A.R. (2023). The Effects of Protein and Carbohydrate Supplementation, with and without Creatine, on Occupational Performance in Firefighters. Nutrients.

[51] Syrotuik D.G., Bell G.J. (2004). Acute creatine monohydrate supplementation: A descriptive physiological profile of responders vs. nonresponders. J. Strength. Cond. Res..

[52] Rawson E.S., Clarkson P. (2000). Acute creatine supplementation in older men. Int. J. Sports Med..

[53] Mihic S., MacDonald J.R., McKenzie S., Tarnopolsky M.A. (2000). Acute creatine loading increases fat-free mass, but does not affect blood pressure, plasma creatinine, or CK activity in men and women. Med. Sci. Sports Exerc..

[54] Jakobi J., Rice C., Curtin S., Marsh G. (2001). Neuromuscular properties and fatigue in older men following acute creatine supplementation. Eur. J. Appl. Physiol..

[55] Kreider R.B., Ferreira M., Wilson M., Grindstaff P., Plisk S., Reinardy J., Cantler E., Almada A. (1998). Effects of creatine supplementation on body composition, strength, and sprint performance. Med. Sci. Sports Exerc..

[56] Cox G., Mujika I., Tumilty D., Burke L. (2002). Acute creatine supplementation and performance during a field test simulating match play in elite female soccer players. Int. J. Sport. Nutr. Exerc. Metab..

[57] Rawson E.S., Volek J.S. (2003). Effects of creatine supplementation and resistance training on muscle strength and weightlifting performance. J. Strength. Cond. Res..

[58] Lanhers C., Pereira B., Naughton G., Trousselard M., Lesage F.X., Dutheil F. (2017). Creatine Supplementation and Upper Limb Strength Performance: A Systematic Review and Meta-Analysis. Sports Med..

[59] Marshall R.P., Droste J.N., Giessing J., Kreider R.B. (2022). Role of Creatine Supplementation in Conditions Involving Mitochondrial Dysfunction: A Narrative Review. Nutrients.

[60] Saks V.A., Kongas O., Vendelin M., Kay L. (2000). Role of the creatine/phosphocreatine system in the regulation of mitochondrial respiration. Acta Physiol. Scand..

[61] Bonilla D.A., Kreider R.B., Stout J.R., Forero D.A., Kerksick C.M., Roberts M.D., Rawson E.S. (2021). Metabolic Basis of Creatine in Health and Disease: A Bioinformatics-Assisted Review. Nutrients.

[62] Nelson A.G., Day R., Glickman-Weiss E.L., Hegsted M., Kokkonen J., Sampson B. (2000). Creatine supplementation alters the response to a graded cycle ergometer test. Eur. J. Appl. Physiol..

[63] Graef J.L., Smith A.E., Kendall K.L., Fukuda D.H., Moon J.R., Beck T.W., Cramer J.T., Stout J.R. (2009). The effects of four weeks of creatine supplementation and high-intensity interval training on cardiorespiratory fitness: A randomized controlled trial. J. Int. Soc. Sports Nutr..

[64] Rossiter H.B., Cannell E.R., Jakeman P.M. (1996). The effect of oral creatine supplementation on the 1000-m performance of competitive rowers. J Sports Sci.

[65] McNaughton L.R., Dalton B., Tarr J. (1998). The effects of creatine supplementation on high-intensity exercise performance in elite performers. Eur. J. Appl. Physiol. Occup. Physiol..

[66] Miura A., Kino F., Kajitani S., Sato H., Fukuba Y. (1999). The effect of oral creatine supplementation on the curvature constant parameter of the power-duration curve for cycle ergometry in humans. Jpn. J. Physiol..

[67] Soares Freitas Sampaio C.R., Aidar F.J., Ferreira A.R.P., Santos J.L.D., Marçal A.C., Matos D.G., Souza R.F., Moreira O.C., Guerra I., Fernandes Filho J. (2020). Can Creatine Supplementation Interfere with Muscle Strength and Fatigue in Brazilian National Level Paralympic Powerlifting?. Nutrients.

[68] Ates O., Keskin B., Bayraktar B. (2017). The Effect of Acute Creatine Supplementation on Fatigue and Anaerobic Performance. Cent. Eur. J. Sport. Sci. Med..

[69] Okudan N., Gokbel H. (2005). The effects of creatine supplementation on performance during the repeated bouts of supramaximal exercise. J. Sports Med. Phys. Fit..

[70] Birch R., Noble D., Greenhaff P.L. (1994). The influence of dietary creatine supplementation on performance during repeated bouts of maximal isokinetic cycling in man. Eur. J. Appl. Physiol. Occup. Physiol..

[71] Machado M., Pereira R., Sampaio-Jorge F., Knifis F., Hackney A. (2009). Creatine supplementation: Effects on blood creatine kinase activity responses to resistance exercise and creatine kinase activity measurement. Braz. J. Pharm. Sci..

[72] Lawler J.M., Barnes W.S., Wu G., Song W., Demaree S. (2002). Direct antioxidant properties of creatine. Biochem. Biophys. Res. Commun..

[73] Jiaming Y., Rahimi M.H. (2021). Creatine supplementation effect on recovery following exercise-induced muscle damage: A systematic review and meta-analysis of randomized controlled trials. J. Food Biochem..

[74] Cooke M.B., Rybalka E., Williams A.D., Cribb P.J., Hayes A. (2009). Creatine supplementation enhances muscle force recovery after eccentrically-induced muscle damage in healthy individuals. J. Int. Soc. Sports Nutr..

[75] van Loon L.J., Murphy R., Oosterlaar A.M., Cameron-Smith D., Hargreaves M., Wagenmakers A.J., Snow R. (2004). Creatine supplementation increases glycogen storage but not GLUT-4 expression in human skeletal muscle. Clin. Sci..

[76] Nelson A.G., Arnall D.A., Kokkonen J., Day R., Evans J. (2001). Muscle glycogen supercompensation is enhanced by prior creatine supplementation. Med. Sci. Sports Exerc..

[77] Forbes S.C., Candow D.G., Neto J.H.F., Kennedy M.D., Forbes J.L., Machado M., Bustillo E., Gomez-Lopez J., Zapata A., Antonio J. (2023). Creatine supplementation and endurance performance: Surges and sprints to win the race. J. Int. Soc. Sports Nutr..

[78] Green A.L., Hultman E., Macdonald I.A., Sewell D.A., Greenhaff P.L. (1996). Carbohydrate ingestion augments skeletal muscle creatine accumulation during creatine supplementation in humans. Am. J. Physiol..

[79] Green A.L., Simpson E.J., Littlewood J.J., Macdonald I.A., Greenhaff P.L. (1996). Carbohydrate ingestion augments creatine retention during creatine feeding in humans. Acta Physiol. Scand..

[80] Rosene J.M., Matthews T.D., McBride K.J., Galla A., Haun M., McDonald K., Gagne N., Lea J., Kasen J., Farias C. (2015). The effects of creatine supplementation on thermoregulation and isokinetic muscular performance following acute (3-day) supplementation. J. Sports Med. Phys. Fit..

[81] Wright G.A., Grandjean P.W., Pascoe D.D. (2007). The effects of creatine loading on thermoregulation and intermittent sprint exercise performance in a hot humid environment. J. Strength. Cond. Res..

[82] Weiss B.A., Powers M.E. (2006). Creatine supplementation does not impair the thermoregulatory response during a bout of exercise in the heat. J. Sports Med. Phys. Fit..

[83] Mendel R.W., Blegen M., Cheatham C., Antonio J., Ziegenfuss T. (2005). Effects of creatine on thermoregulatory responses while exercising in the heat. Nutrition.

[84] Rosene J.M., Whitman S.A., Fogarty T.D. (2004). A Comparison of Thermoregulation with Creatine Supplementation between the Sexes in a Thermoneutral Environment. J. Athl. Train..

[85] Kelly V.G., Jenkins D.G. (1998). Effect of oral creatine supplementation on near-maximal strength and repeated sets of high-intensity bench press exercise. J. Strength. Cond. Res..

[86] Chrusch M.J., Chilibeck P.D., Chad K.E., Davison K.S., Burke D.G. (2001). Creatine supplementation combined with resistance training in older men. Med. Sci. Sports Exerc..

[87] Bemben M.G., Bemben D.A., Loftiss D.D., Knehans A.W. (2001). Creatine supplementation during resistance training in college football athletes. Med. Sci. Sports Exerc..

[88] Syrotuik D.G., Bell G.J., Burnham R., Sim L.L., Calvert R.A., Maclean I.M. (2000). Absolute and relative strength performance following creatine monohydrate supplementation combined with periodized resistance training. J. Strength. Cond. Res..

[89] Larson-Meyer D.E., Hunter G.R., Trowbridge C.A., Turk J.C., Ernest J.M., Torman S.L., Harbin P.A. (2000). The effect of creatine supplementation on muscle strength and body composition during off-season training in female soccer players. J. Strength. Cond. Res..

[90] Theodorou A.S., Cooke C.B., King R.F., Hood C., Denison T., Wainwright B.G., Havenetidis K. (1999). The effect of longer-term creatine supplementation on elite swimming performance after an acute creatine loading. J. Sports Sci..

[91] Hopwood M.J., Graham K., Rooney K.B. (2006). Creatine supplementation and swim performance: A brief review. J. Sports Sci. Med..

[92] Bennett T., Bathalon G., Armstrong III D., Martin B., Coll R., Beck R., Barkdull T., O’Brien K., Deuster P.A. (2001). Effect of creatine on performance of militarily relevant tasks and soldier health. Mil. Med..

[93] Volek J.S., Duncan N.D., Mazzetti S.A., Staron R.S., Putukian M., Gómez A.L., Pearson D.R., Fink W.J., Kraemer W.J. (1999). Performance and muscle fiber adaptations to creatine supplementation and heavy resistance training. Med. Sci. Sports Exerc..

[94] Kendall K.L., Smith A.E., Graef J.L., Fukuda D.H., Moon J.R., Beck T.W., Cramer J.T., Stout J.R. (2009). Effects of four weeks of high-intensity interval training and creatine supplementation on critical power and anaerobic working capacity in college-aged men. J. Strength. Cond. Res..

[95] Hickner R.C., Dyck D.J., Sklar J., Hatley H., Byrd P. (2010). Effect of 28 days of creatine ingestion on muscle metabolism and performance of a simulated cycling road race. J. Int. Soc. Sports Nutr..

[96] da Silveira C.L., de Souza T.S., Batista G.R., de Araújo A.T., da Silva J.C., de Sousa Mdo S., Marta C., Garrido N.D. (2014). Is long term creatine and glutamine supplementation effective in enhancing physical performance of military police officers?. J. Hum. Kinet..

[97] Warber J.P., Tharion W.J., Patton J.F., Champagne C.M., Mitotti P., Lieberman H.R. (2002). The effect of creatine monohydrate supplementation on obstacle course and multiple bench press performance. J. Strength. Cond. Res..

[98] Poston W.S., Haddock C.K., Jahnke S.A., Jitnarin N., Tuley B.C., Kales S.N. (2011). The prevalence of overweight, obesity, and substandard fitness in a population-based firefighter cohort. J. Occup. Env. Med..

[99] Jagim A.R., Luedke J.A., Dobbs W.C., Almonroeder T., Markert A., Zapp A., Askow A.T., Kesler R.M., Fields J.B., Jones M.T. (2023). Physiological Demands of a Self-Paced Firefighter Air-Management Course and Determination of Work Efficiency. J. Funct. Morphol. Kinesiol..

[100] Aragon A.A., Schoenfeld B.J., Wildman R., Kleiner S., VanDusseldorp T., Taylor L., Earnest C.P., Arciero P.J., Wilborn C., Kalman D.S. (2017). International society of sports nutrition position stand: Diets and body composition. J. Int. Soc. Sports Nutr..

[101] Forbes S.C., Candow D.G., Ostojic S.M., Roberts M.D., Chilibeck P.D. (2021). Meta-analysis examining the importance of creatine ingestion strategies on lean tissue mass and strength in older adults. Nutrients.

[102] Forbes S.C., Candow D.G., Krentz J.R., Roberts M.D., Young K.C. (2019). Changes in fat mass following creatine supplementation and resistance training in adults≥ 50 years of age: A meta-analysis. J. Funct. Morphol. Kinesiol..

[103] Delpino F.M., Figueiredo L.M., Forbes S.C., Candow D.G., Santos H.O. (2022). Influence of age, sex, and type of exercise on the efficacy of creatine supplementation on lean body mass: A systematic review and meta-analysis of randomized clinical trials. Nutrition.

[104] Chilibeck P.D., Kaviani M., Candow D.G., Zello G.A. (2017). Effect of creatine supplementation during resistance training on lean tissue mass and muscular strength in older adults: A meta-analysis. Open Access J. Sports Med..

[105] Candow D.G., Chilibeck P.D., Forbes S.C. (2014). Creatine supplementation and aging musculoskeletal health. Endocrine.

[106] Burke R., Piñero A., Coleman M., Mohan A., Sapuppo M., Augustin F., Aragon A.A., Candow D.G., Forbes S.C., Swinton P. (2023). The effects of creatine supplementation combined with resistance training on regional measures of muscle hypertrophy: A systematic review with meta-analysis. Nutrients.

[107] Devries M.C., Phillips S.M. (2014). Creatine supplementation during resistance training in older adults—A meta-analysis. Med. Sci. Sports Exerc..

[108] Kazak L., Rahbani J.F., Samborska B., Lu G.Z., Jedrychowski M.P., Lajoie M., Zhang S., Ramsay L., Dou F.Y., Tenen D. (2019). Ablation of adipocyte creatine transport impairs thermogenesis and causes diet-induced obesity. Nat. Metab..

[109] Kazak L., Chouchani E.T., Lu G.Z., Jedrychowski M.P., Bare C.J., Mina A.I., Kumari M., Zhang S., Vuckovic I., Laznik-Bogoslavski D. (2017). Genetic depletion of adipocyte creatine metabolism inhibits diet-induced thermogenesis and drives obesity. Cell Metab..

[110] Kazak L., Chouchani E.T., Jedrychowski M.P., Erickson B.K., Shinoda K., Cohen P., Vetrivelan R., Lu G.Z., Laznik-Bogoslavski D., Hasenfuss S.C. (2015). A creatine-driven substrate cycle enhances energy expenditure and thermogenesis in beige fat. Cell.

[111] Forbes S.C., Ostojic S.M., Souza-Junior T.P., Candow D.G. (2022). A High Dose of Creatine Combined with Resistance Training Appears to Be Required to Augment Indices of Bone Health in Older Adults. Ann. Nutr. Metab..

[112] Chilibeck P.D., Candow D.G., Landeryou T., Kaviani M., Paus-Jenssen L. (2015). Effects of creatine and resistance training on bone health in postmenopausal women. Med. Sci. Sports Exerc..

[113] Chilibeck P., Chrusch M., Chad K., Davison K.S., Burke D. (2005). Creatine monohydrate and resistance training increase bone mineral content and density in older men. J. Nutr. Health Aging.

[114] Candow D.G., Chilibeck P.D., Gordon J., Vogt E., Landeryou T., Kaviani M., Paus-Jensen L. (2021). Effect of 12 months of creatine supplementation and whole-body resistance training on measures of bone, muscle and strength in older males. Nutr. Health.

[115] Forbes S.C., Chilibeck P.D., Candow D.G. (2018). Creatine supplementation during resistance training does not lead to greater bone mineral density in older humans: A brief meta-analysis. Front. Nutr..

[116] Wallimann T., Tokarska-Schlattner M., Schlattner U. (2011). The creatine kinase system and pleiotropic effects of creatine. Amino Acids.

[117] Chilibeck P.D., Candow D.G., Gordon J.J., Duff W.R.D., Mason R., Shaw K., Taylor-Gjevre R., Nair B., Zello G.A. (2023). A 2-yr Randomized Controlled Trial on Creatine Supplementation during Exercise for Postmenopausal Bone Health. Med. Sci. Sports Exerc..

[118] McAllister M.J., Basham S.A., Smith J.W., Waldman H.S., Krings B.M., Mettler J.A., Butawan M.B., Bloomer R.J. (2018). Effects of environmental heat and antioxidant ingestion on blood markers of oxidative stress in professional firefighters performing structural fire exercises. J. Occup. Environ. Med..

[119] Clarke H., Kim D.-H., Meza C.A., Ormsbee M.J., Hickner R.C. (2020). The Evolving Applications of Creatine Supplementation: Could Creatine Improve Vascular Health?. Nutrients.

[120] Clarke H., Hickner R.C., Ormsbee M.J. (2021). The Potential Role of Creatine in Vascular Health. Nutrients.

[121] Balestrino M. (2021). Role of Creatine in the Heart: Health and Disease. Nutrients.

[122] Arciero P.J., Hannibal N.S., Nindl B.C., Gentile C.L., Hamed J., Vukovich M.D. (2001). Comparison of creatine ingestion and resistance training on energy expenditure and limb blood flow. Metabolism.

[123] Sanchez-Gonzalez M.A., Wieder R., Kim J.-S., Vicil F., Figueroa A. (2011). Creatine supplementation attenuates hemodynamic and arterial stiffness responses following an acute bout of isokinetic exercise. Eur. J. Appl. Physiol..

[124] Aubry R.L., Whinton A.K., Burr J.F. (2018). The effect of creatine supplementation on the response of central and peripheral pulse wave velocity to high-intensity resistance exercise. Cogent Med..

[125] Pellinger T.K., Gimblet C.J., Vance M.M., Shepherd M., Ortlip A.T., Staudmyer T.B., Lamanca J.J., Werner T.J. (2020). Effect of Acute Creatine Supplementation on Arterial Stiffness and Muscle Oxygen Saturation. FASEB J..

[126] de Moraes R., Van Bavel D., de Moraes B.S., Tibiriçá E. (2014). Effects of dietary creatine supplementation on systemic microvascular density and reactivity in healthy young adults. Nutr. J..

[127] Van Bavel D., de Moraes R., Tibirica E. (2019). Effects of dietary supplementation with creatine on homocysteinemia and systemic microvascular endothelial function in individuals adhering to vegan diets. Fundam. Clin. Pharmacol..

[128] Earnest C.P., Almada A.L., Mitchell T.L. (1996). High-performance capillary electrophoresis-pure creatine monohydrate reduces blood lipids in men and women. Clin. Sci..

[129] Meyer L.E., Machado L.B., Santiago A.P.S., da-Silva W.S., De Felice F.G., Holub O., Oliveira M.F., Galina A. (2006). Mitochondrial creatine kinase activity prevents reactive oxygen species generation: Antioxidant role of mitochondrial kinase-dependent ADP re-cycling activity. J. Biol. Chem..

[130] Korzun W.J. (2004). Oral creatine supplements lower plasma homocysteine concentrations in humans. Am. Soc. Clin. Lab. Sci..

[131] Sestili P., Martinelli C., Bravi G., Piccoli G., Curci R., Battistelli M., Falcieri E., Agostini D., Gioacchini A.M., Stocchi V. (2006). Creatine supplementation affords cytoprotection in oxidatively injured cultured mammalian cells via direct antioxidant activity. Free Radic. Biol. Med..

[132] Matthews R.T., Yang L., Jenkins B.G., Ferrante R.J., Rosen B.R., Kaddurah-Daouk R., Beal M.F. (1998). Neuroprotective effects of creatine and cyclocreatine in animal models of Huntington’s disease. J. Neurosci..

[133] Kingsley M., Cunningham D., Mason L., Kilduff L.P., McEneny J. (2009). Role of creatine supplementation on exercise-induced cardiovascular function and oxidative stress. Oxid. Med. Cell Longev..

[134] Rahimi R. (2011). Creatine supplementation decreases oxidative DNA damage and lipid peroxidation induced by a single bout of resistance exercise. J. Strength. Cond. Res..

[135] Amiri E., Sheikholeslami-Vatani D. (2023). The role of resistance training and creatine supplementation on oxidative stress, antioxidant defense, muscle strength, and quality of life in older adults. Front. Public. Health.

[136] Percário S., Domingues S.P., Teixeira L.F., Vieira J.L., de Vasconcelos F., Ciarrocchi D.M., Almeida E.D., Conte M. (2012). Effects of creatine supplementation on oxidative stress profile of athletes. J. Int. Soc. Sports Nutr..

[137] Ji L., Zhao X., Zhang B., Kang L., Song W., Zhao B., Xie W., Chen L., Hu X. (2019). Slc6a8-Mediated Creatine Uptake and Accumulation Reprogram Macrophage Polarization via Regulating Cytokine Responses. Immunity.

[138] Di Biase S., Ma X., Wang X., Yu J., Wang Y.C., Smith D.J., Zhou Y., Li Z., Kim Y.J., Clarke N. (2019). Creatine uptake regulates CD8 T cell antitumor immunity. J. Exp. Med..

[139] Bredahl E.C., Eckerson J.M., Tracy S.M., McDonald T.L., Drescher K.M. (2021). The Role of Creatine in the Development and Activation of Immune Responses. Nutrients.

[140] Almeida F.M., Battochio A.S., Napoli J.P., Alves K.A., Balbin G.S., Oliveira-Junior M., Moriya H.T., Pego-Fernandes P.M., Vieira R.P., Pazetti R. (2020). Creatine Supply Attenuates Ischemia-Reperfusion Injury in Lung Transplantation in Rats. Nutrients.

[141] Santos R.V., Bassit R.A., Caperuto E.C., Costa Rosa L.F. (2004). The effect of creatine supplementation upon inflammatory and muscle soreness markers after a 30 km race. Life Sci..

[142] Bassit R., Curi R., Costa Rosa L.F.B.P. (2008). Creatine supplementation reduces plasma levels of pro-inflammatory cytokines and PGE 2 after a half-ironman competition. Amino Acids.

[143] Rawson E.S., Conti M.P., Miles M.P. (2007). Creatine supplementation does not reduce muscle damage or enhance recovery from resistance exercise. J. Strength. Cond. Res..

[144] Oliveira C.L., Antunes B.d.M.M., Gomes A.C., Lira F.S., Pimentel G.D., Boulé N.G., Mota J.F. (2020). Creatine supplementation does not promote additional effects on inflammation and insulin resistance in older adults: A pilot randomized, double-blind, placebo-controlled trial. Clin. Nutr. ESPEN.

[145] Lindberg A.S., Oksa J., Gavhed D., Malm C. (2013). Field tests for evaluating the aerobic work capacity of firefighters. PLoS ONE.

[146] Horn G.P., Blevins S., Fernhall B., Smith D.L. (2013). Core temperature and heart rate response to repeated bouts of firefighting activities. Ergonomics.

[147] Gledhill N., Jamnik V.K. (1992). Characterization of the physical demands of firefighting. Can. J. Sport. Sci..

[148] Taylor N.A., Lewis M.C., Notley S.R., Peoples G.E. (2012). A fractionation of the physiological burden of the personal protective equipment worn by firefighters. Eur. J. Appl. Physiol..

[149] Lesniak A.Y., Bergstrom H.C., Clasey J.L., Stromberg A.J., Abel M.G. (2020). The Effect of Personal Protective Equipment on Firefighter Occupational Performance. J. Strength. Cond. Res..

[150] Eves N.D., Jones R.L., Petersen S.R. (2005). The influence of the self-contained breathing apparatus (SCBA) on ventilatory function and maximal exercise. Can. J. Appl. Physiol..

[151] Rossi R. (2003). Fire fighting and its influence on the body. Ergonomics.

[152] Smith D.L., Petruzzello S., Kramer J., Misner J. (1997). The effects of different thermal environments on the physiological and psychological responses of firefighters to a training drill. Ergonomics.

[153] Colburn D., Suyama J., Reis S.E., Morley J.L., Goss F.L., Chen Y.-F., Moore C.G., Hostler D. (2011). A comparison of cooling techniques in firefighters after a live burn evolution. Prehospital Emerg. Care.

[154] Volek J.S., Mazzetti S.A., Farquhar W.B., Barnes B.R., Gómez A.L., Kraemer W.J. (2001). Physiological responses to short-term exercise in the heat after creatine loading. Med. Sci. Sports Exerc..

[155] Kern M., Podewils L.J., Vukovich M., Buono M.J. (2001). Physiological response to exercise in the heat following creatine supplementation. J. Exerc. Physiol. Online.

[156] Dalbo V.J., Roberts M.D., Stout J.R., Kerksick C.M. (2008). Putting to rest the myth of creatine supplementation leading to muscle cramps and dehydration. Br. J. Sports Med..

[157] Lopez R.M., Casa D.J., McDermott B.P., Ganio M.S., Armstrong L.E., Maresh C.M. (2009). Does creatine supplementation hinder exercise heat tolerance or hydration status? A systematic review with meta-analyses. J. Athl. Train..

[158] Branch J.D., Schwarz W.D., Van Lunen B. (2007). Effect of creatine supplementation on cycle ergometer exercise in a hyperthermic environment. J. Strength. Cond. Res..

[159] Easton C., Turner S., Pitsiladis Y.P. (2007). Creatine and glycerol hyperhydration in trained subjects before exercise in the heat. Int. J. Sport. Nutr. Exerc. Metab..

[160] Watson G., Casa D.J., Fiala K.A., Hile A., Roti M.W., Healey J.C., Armstrong L.E., Maresh C.M. (2006). Creatine use and exercise heat tolerance in dehydrated men. J. Athl. Train..

[161] Oopik V., Paasuke M., Timpmann S., Medijainen L., Ereline J., Gapejeva J. (2002). Effects of creatine supplementation during recovery from rapid body mass reduction on metabolism and muscle performance capacity in well-trained wrestlers. J. Sports Med. Phys. Fit..

[162] DeMoulin D., Jacobs S., Nam Y.-S., Harding A.B., Moskowitz A.F., Shi Y., Kim H. (2022). Mental health among firefighters: Understanding the mental health risks, treatment barriers, and coping strategies. J. Occup. Environ. Med..

[163] Katsavouni F., Bebetsos E., Malliou P., Beneka A. (2016). The relationship between burnout, PTSD symptoms and injuries in firefighters. Occup. Med..

[164] Skogstad M., Skorstad M., Lie A., Conradi H.S., Heir T., Weisæth L. (2013). Work-related post-traumatic stress disorder. Occup. Med..

[165] Henderson S.N., Van Hasselt V.B., LeDuc T.J., Couwels J. (2016). Firefighter suicide: Understanding cultural challenges for mental health professionals. Prof. Psychol. Res. Pract..

[166] Stanley I.H., Hom M.A., Hagan C.R., Joiner T.E. (2015). Career prevalence and correlates of suicidal thoughts and behaviors among firefighters. J. Affect. Disord..

[167] Stanley I.H., Boffa J.W., Hom M.A., Kimbrel N.A., Joiner T.E. (2017). Differences in psychiatric symptoms and barriers to mental health care between volunteer and career firefighters. Psychiatry Res..

[168] Allen P.J., D’Anci K.E., Kanarek R.B., Renshaw P.F. (2012). Sex-specific antidepressant effects of dietary creatine with and without sub-acute fluoxetine in rats. Pharmacol. Biochem. Behav..

[169] Kondo D.G., Forrest L.N., Shi X., Sung Y.H., Hellem T.L., Huber R.S., Renshaw P.F. (2016). Creatine target engagement with brain bioenergetics: A dose-ranging phosphorus-31 magnetic resonance spectroscopy study of adolescent females with SSRI-resistant depression. Amino Acids.

[170] Bender A., Klopstock T. (2016). Creatine for neuroprotection in neurodegenerative disease: End of story?. Amino Acids.

[171] Jagim A.R., Kerksick C.M. (2021). Creatine Supplementation in Children and Adolescents. Nutrients.

[172] Brosnan J.T., Brosnan M.E. (2007). Creatine: Endogenous metabolite, dietary, and therapeutic supplement. Annu. Rev. Nutr..

[173] Rae C., Digney A.L., McEwan S.R., Bates T.C. (2003). Oral creatine monohydrate supplementation improves brain performance: A double-blind, placebo-controlled, cross-over trial. Proc. Biol. Sci..

[174] McMorris T., Mielcarz G., Harris R.C., Swain J.P., Howard A. (2007). Creatine supplementation and cognitive performance in elderly individuals. Neuropsychol. Dev. Cogn. B Aging Neuropsychol. Cogn..

[175] Wolffe T.A., Robinson A., Clinton A., Turrell L., Stec A.A. (2023). Mental health of UK firefighters. Sci. Rep..

[176] Van Hasselt V.B., Bourke M.L., Schuhmann B.B. (2022). Firefighter Stress and Mental Health: Introduction to the Special Issue. Behav. Modif..

[177] Kauffman B., Manning K., Zvolensky M.J., Vujanovic A.A. (2022). Fatigue Sensitivity and Mental Health among Trauma-Exposed Firefighters. Fatigue.

[178] Harvey S.B., Milligan-Saville J.S., Paterson H.M., Harkness E.L., Marsh A.M., Dobson M., Kemp R., Bryant R.A. (2016). The mental health of fire-fighters: An examination of the impact of repeated trauma exposure. Aust. N. Z. J. Psychiatry.

[179] MacDermid J.C., Lomotan M., Hu M.A. (2021). Canadian Career Firefighters’ Mental Health Impacts and Priorities. Int. J. Environ. Res. Public. Health.

[180] Hom M.A., Stanley I.H., Spencer-Thomas S., Joiner T.E. (2018). Mental health service use and help-seeking among women firefighters with a career history of suicidality. Psychol. Serv..

[181] Dextras-Gauthier J., Gilbert M.H., Dima J., Adou L.B. (2023). Organizational culture and leadership behaviors: Is manager’s psychological health the missing piece?. Front. Psychol..

[182] Counson I., Hosemans D., Lal T.J., Mott B., Harvey S.B., Joyce S. (2019). Mental health and mindfulness amongst Australian fire fighters. BMC Psychol..

[183] Senger A.R., McGrew S.J., Gallagher M.W., Vujanovic A. (2023). Associations of resilience and hope with mental and physical health among firefighters. J. Clin. Psychol..

[184] Roitman S., Green T., Osher Y., Karni N., Levine J. (2007). Creatine monohydrate in resistant depression: A preliminary study. Bipolar Disord..

[185] Ortega M.A., Fraile-Martínez Ó., García-Montero C., Alvarez-Mon M.A., Lahera G., Monserrat J., Llavero-Valero M., Gutiérrez-Rojas L., Molina R., Rodríguez-Jimenez R. (2022). Biological Role of Nutrients, Food and Dietary Patterns in the Prevention and Clinical Management of Major Depressive Disorder. Nutrients.

[186] Bakian A.V., Huber R.S., Scholl L., Renshaw P.F., Kondo D. (2020). Dietary creatine intake and depression risk among U.S. adults. Transl. Psychiatry.

[187] Gabriel F.C., Oliveira M., Martella B.D.M., Berk M., Brietzke E., Jacka F.N., Lafer B. (2023). Nutrition and bipolar disorder: A systematic review. Nutr. Neurosci..

[188] Kious B.M., Sabic H., Sung Y.-H., Kondo D.G., Renshaw P. (2017). An open-label pilot study of combined augmentation with creatine monohydrate and 5-hydroxytryptophan for selective serotonin reuptake inhibitor–or serotonin-norepinephrine reuptake inhibitor–resistant depression in adult women. J. Clin. Psychopharmacol..

[189] Nemets B., Levine J. (2013). A pilot dose-finding clinical trial of creatine monohydrate augmentation to SSRIs/SNRIs/NASA antidepressant treatment in major depression. Int. Clin. Psychopharmacol..

[190] Hellem T.L., Sung Y.-H., Shi X.-F., Pett M.A., Latendresse G., Morgan J., Huber R.S., Kuykendall D., Lundberg K.J., Renshaw P.F. (2015). A pilot study of creatine as a novel treatment for depression in methamphetamine using females. J. Dual Diagn..

[191] Skarupski K.A., Tangney C., Li H., Ouyang B., Evans D.A., Morris M.C. (2010). Longitudinal association of vitamin B-6, folate, and vitamin B-12 with depressive symptoms among older adults over time. Am. J. Clin. Nutr..

[192] Avgerinos K.I., Spyrou N., Bougioukas K.I., Kapogiannis D. (2018). Effects of creatine supplementation on cognitive function of healthy individuals: A systematic review of randomized controlled trials. Exp. Gerontol..

[193] Bender A., Samtleben W., Elstner M., Klopstock T. (2008). Long-term creatine supplementation is safe in aged patients with Parkinson disease. Nutr. Res..

[194] Andreassen O.A., Dedeoglu A., Ferrante R.J., Jenkins B.G., Ferrante K.L., Thomas M., Friedlich A., Browne S.E., Schilling G., Borchelt D.R. (2001). Creatine increases survival and delays motor symptoms in a transgenic animal model of Huntington’s disease. Neurobiol. Dis..

[195] Hersch S.M., Schifitto G., Oakes D., Bredlau A.-L., Meyers C.M., Nahin R., Rosas H.D., Investigators H.S.G.C.-E., Huntington Study Group CREST-E Investigators and Coordinators (2017). The CREST-E study of creatine for Huntington disease: A randomized controlled trial. Neurology.

[196] Allen P.J., D’anci K.E., Kanarek R.B., Renshaw P.F. (2010). Chronic creatine supplementation alters depression-like behavior in rodents in a sex-dependent manner. Neuropsychopharmacology.

[197] Pazini F.L., Cunha M.P., Rosa J.M., Colla A.R., Lieberknecht V., Oliveira Á., Rodrigues A.L.S. (2016). Creatine, similar to ketamine, counteracts depressive-like behavior induced by corticosterone via PI3K/Akt/mTOR pathway. Mol. Neurobiol..

[198] Ahn N., Leem Y.H., Kato M., Chang H. (2016). Effects of creatine monohydrate supplementation and exercise on depression-like behaviors and raphe 5-HT neurons in mice. J. Exerc. Nutr. Biochem..

[199] Collias N. Congress Pushes DOD to Consider Adding Creatine to Military Rations; Natural Products Insider: 2024; Volume 2024. https://www.supplysidesj.com/sports-nutrition/congress-pushes-dod-to-consider-adding-creatine-to-military-rations.

[200] Ohtsuki S., Tachikawa M., Takanaga H., Shimizu H., Watanabe M., Hosoya K.-i., Terasaki T. (2002). The blood–brain barrier creatine transporter is a major pathway for supplying creatine to the brain. J. Cereb. Blood Flow. Metab..

[201] Smith R.N., Agharkar A.S., Gonzales E.B. (2014). A review of creatine supplementation in age-related diseases: More than a supplement for athletes. F1000Res.

[202] Béard E., Braissant O. (2010). Synthesis and transport of creatine in the CNS: Importance for cerebral functions. J. Neurochem..

[203] Andres R.H., Ducray A.D., Schlattner U., Wallimann T., Widmer H.R. (2008). Functions and effects of creatine in the central nervous system. Brain Res. Bull..

[204] Ostojic S.M., Ostojic J., Drid P., Vranes M. (2016). Guanidinoacetic acid versus creatine for improved brain and muscle creatine levels: A superiority pilot trial in healthy men. Appl. Physiol. Nutr. Metab..

[205] ME B., HK B., JG G. (1952). Betaine and glycocyamine in the treatment of disability resulting from acute anterior poliomyelitis. Ann. West. Med. Surg..

[206] Sakellaris G., Partalis N., Nasis G., Kotsiou M., Tamiolaki M., Bouloukaki E., Evangeliou A. (2012). Outcome of traumatic dysarthria and lingual problems of understanding with creatine administration. an open label randomized pilot study. J. Trauma. Treat..

[207] Sakellaris G., Kotsiou M., Tamiolaki M., Kalostos G., Tsapaki E., Spanaki M., Spilioti M., Charissis G., Evangeliou A. (2006). Prevention of complications related to traumatic brain injury in children and adolescents with creatine administration: An open label randomized pilot study. J. Trauma..

[208] Ainsley Dean P.J., Arikan G., Opitz B., Sterr A. (2017). Potential for use of creatine supplementation following mild traumatic brain injury. Concussion.

[209] Uehli K., Mehta A.J., Miedinger D., Hug K., Schindler C., Holsboer-Trachsler E., Leuppi J.D., Künzli N. (2014). Sleep problems and work injuries: A systematic review and meta-analysis. Sleep. Med. Rev..

[210] Patterson P.D., Weaver M.D., Frank R.C., Warner C.W., Martin-Gill C., Guyette F.X., Fairbanks R.J., Hubble M.W., Songer T.J., Callaway C.W. (2012). Association between poor sleep, fatigue, and safety outcomes in emergency medical services providers. Prehosp Emerg. Care.

[211] Marvin G., Schram B., Orr R., Canetti E.F.D. (2023). Occupation-Induced Fatigue and Impacts on Emergency First Responders: A Systematic Review. Int. J. Environ. Res. Public. Health.

[212] Brinkman J.E., Reddy V., Sharma S. (2024). Physiology of Sleep. StatPearls [Internet].

[213] Scharf M.T., Naidoo N., Zimmerman J.E., Pack A.I. (2008). The energy hypothesis of sleep revisited. Prog. Neurobiol..

[214] Harris J.J., Jolivet R., Attwell D. (2012). Synaptic energy use and supply. Neuron.

[215] Schlattner U., Klaus A., Ramirez Rios S., Guzun R., Kay L., Tokarska-Schlattner M. (2016). Cellular compartmentation of energy metabolism: Creatine kinase microcompartments and recruitment of B-type creatine kinase to specific subcellular sites. Amino Acids.

[216] Dechent P., Pouwels P., Wilken B., Hanefeld F., Frahm J. (1999). Increase of total creatine in human brain after oral supplementation of creatine-monohydrate. Am. J. Physiol.-Regul. Integr. Comp. Physiol..

[217] Gordji-Nejad A., Matusch A., Kleedörfer S., Jayeshkumar Patel H., Drzezga A., Elmenhorst D., Binkofski F., Bauer A. (2024). Single dose creatine improves cognitive performance and induces changes in cerebral high energy phosphates during sleep deprivation. Sci. Rep..

[218] Dworak M., Kim T., Mccarley R.W., Basheer R. (2017). Creatine supplementation reduces sleep need and homeostatic sleep pressure in rats. J. Sleep. Res..

[219] Buford T.W., Kreider R.B., Stout J.R., Greenwood M., Campbell B., Spano M., Ziegenfuss T., Lopez H., Landis J., Antonio J. (2007). International Society of Sports Nutrition position stand: Creatine supplementation and exercise. J. Int. Soc. Sports Nutr..

[220] Greenwood M., Kreider R.B., Greenwood L., Byars A. (2003). Cramping and injury incidence in collegiate football players are reduced by creatine supplementation. J. Athl. Train..

[221] Kreider R.B., Melton C., Rasmussen C.J., Greenwood M., Lancaster S., Cantler E.C., Milnor P., Almada A.L. (2003). Long-term creatine supplementation does not significantly affect clinical markers of health in athletes. Mol. Cell Biochem..

[222] Rodriguez N.R., DiMarco N.M., Langley S., American Dietetic Association, Dietitians of Canada, American College of Sports Medicine: Nutrition and Athletic Performance (2009). Position of the American Dietetic Association, Dietitians of Canada, and the American College of Sports Medicine: Nutrition and athletic performance. J. Am. Diet. Assoc..

[223] Persky A.M., Rawson E.S. (2007). Safety of creatine supplementation. Creatine and Creatine Kinase in Health and Disease.

[224] Gualano B., de Salles Painelli V., Roschel H., Lugaresi R., Dorea E., Artioli G.G., Lima F.R., da Silva M.E., Cunha M.R., Seguro A.C. (2011). Creatine supplementation does not impair kidney function in type 2 diabetic patients: A randomized, double-blind, placebo-controlled, clinical trial. Eur. J. Appl. Physiol..

[225] Ostojic S.M., Grasaas E., Cvejic J. (2023). Dietary creatine and cancer risk in the U.S. population: NHANES 2017–2020. J. Funct. Foods.

[226] Ostojic S.M., Stea T.H., Ellery S.J., Smith-Ryan A.E. (2024). Association between dietary intake of creatine and female reproductive health: Evidence from NHANES 2017–2020. Food Sci. Nutr..

[227] Ostojic S.M. (2021). Dietary creatine and kidney function in adult population: NHANES 2017-2018. Food Sci. Nutr..

[228] Zhou B., Hong M., Jin L., Ling K. (2024). Exploring the relationship between creatine supplementation and renal function: Insights from Mendelian randomization analysis. Ren. Fail..

